# Childhood BMI and other measures of body composition as a predictor of cardiometabolic non-communicable diseases in adulthood: a systematic review

**DOI:** 10.1017/S136898002200235X

**Published:** 2022-10-24

**Authors:** Amela Bander, Alexia J Murphy-Alford, Victor O Owino, Cornelia U Loechl, Jonathan CK Wells, Imara Gluning, Marko Kerac

**Affiliations:** 1 Department of Population Health, London School of Hygiene and Tropical Medicine, London WC1E 7HT, UK; 2 Nutritional and Health Related Environmental Studies Section, Division of Human Health, International Atomic Energy Agency, Vienna, Austria; 3 Population, Policy and Practice Research Teaching Department, University College London, London, UK; 4 Brighton and Sussex University Hospitals Trust, Brighton, UK; 5 Centre for Maternal, Adolescent, Reproductive & Child Health (MARCH), London School of Hygiene & Tropical Medicine, London, UK

**Keywords:** Body composition, Non-communicable diseases, BMI, Cardio-metabolic health

## Abstract

**Objective:**

There is growing evidence that childhood malnutrition is associated with non-communicable diseases (NCD) in adulthood and that body composition mediates some of this association. This review aims to determine if childhood body composition can be used to predict later-life cardiometabolic NCD and which measures of body composition predicts future NCD.

**Design:**

Electronic databases were searched for articles where: children aged under 5 years had body composition measured; cardiometabolic health outcomes were measured a minimum of 10 years later.

**Setting:**

The databases Embase, Medline and Global Health were searched through July 2020.

**Participants:**

Children aged under 5 years with a follow-up of minimum 10 years.

**Results:**

Twenty-nine studies met the inclusion criteria. Though a poor proxy measure of body composition, body mass index (BMI) was commonly reported (*n* 28, 97 %). 25 % of these studies included an additional measure (ponderal index or skinfold thickness). Few studies adjusted for current body size (*n* 11, 39 %).

**Conclusions:**

Many studies reported that low infant BMI and high childhood BMI were associated with an increased risk of NCD-related outcomes in later life but no conclusions can be made about the exact timing of child malnutrition and consequent impact on NCD. Because studies focussed on BMI rather than direct measures of body composition, nothing can be said about which measures of body composition in childhood are most useful. Future research on child nutrition and long-term outcomes is urgently needed and should include validated body composition assessments as well as standard anthropometric and BMI measurements.

Non-communicable diseases (NCD), such as cardiovascular diseases (CVD), diabetes and chronic respiratory diseases, are the leading cause of mortality, equivalent to 71 % of deaths worldwide, and are projected to increase even further, reaching 52 million deaths by 2030^(1)^.

Risk factors for NCD include both social factors (poverty, education and stress) and biological factors (e.g. genetic predisposition; foetal epigenetic changes with life-course consequence): the former highly affects lifestyle factors such as diet and physical activity^(2)^. Early life malnutrition, which in this review is defined as the first 5 years of postnatal life, is also a key risk factor for NCD and refers to insufficient energy- and/or nutrient intake; but also refers to an excessive and imbalanced energy intake, often resulting in overweight or obesity^(3)^. For assessing nutritional status in children and adults, anthropometric indicators of growth and body size such as weight-for-height (WHZ), weight-for-age (WAZ), BMI and mid-upper arm circumference (MUAC) amongst others are commonly used^(4)^. However, there is growing evidence that anthropometry alone has limitations in describing nutrition-related risk (of morbidity/mortality)^(5)^. Body composition measures are attracting interest as potentially much better indicators of both short-^(6)^ and long-term risk^(7,8)^. Measures of body composition vary from those related to anthropometry, e.g waist circumference (WC); waist-hip ratio and skinfold (SF) thickness, to indirect measures such as bioelectrical impedance analysis (BIA) to more direct measures such as dual-energy X-ray absorptiometry (DXA/DEXA) scan, isotope dilution or densitometry^(9)^.

There is extensive evidence that exposure to *in-utero* undernutrition increases the risk of NCD in later life^(10–12)^ and that being overweight in adulthood also increases the risk of NCD^(13,14)^. There is also emerging evidence relating to childhood exposures^(15)^, one recent review found that ‘*exposure to severe malnutrition or famine in childhood was consistently associated with increased risk of CVD, hypertension, impaired glucose metabolism and metabolic syndrome in later life*’^(16)^. In attempts to better understand the link between such episodes of early-life malnutrition to later-life health and NCD, an increasing number of studies are assessing body composition in childhood^(17,18)^. Whilst plausible^(19)^, the links between body composition in early life and later-life NCD are not currently well understood^(20–25)^. Moreover, this linkage has not been evaluated through a systematic review: previous work focuses on early-life anthropmetry and NCD rather than body composition and NCD. This represents a major evidence gap, since anthropometry alone is a relatively crude measure of nutrition and growth. It does not, for example, reflect the fact that two similar-sized individuals can have very different percentages of underlying fat and muscle mass^(26)^. This matters because both fat and muscle are metabolically active organs and have a bearing on an individuals’ physiology, metabolism and in turn risks of health and disease. Hence, understanding body composition in early childhood rather than body size alone may transform our understanding of the mechanisms by which early undernutrition affects later life NCD risk. Such understanding is particularly important to those in the global child nutrition community where a traditional focus of malnutrition treatment programmes has been on regaining as much weight as quickly as possible. This may have implications for short-term body composition^(18)^ and in turn for long-term adult NCD risk. Potential policy implications include greater focus on *healthy* growth rather than just growth alone in programmes managing child malnutrition.

Our review thus aims to synthesise evidence on early life body composition and long-term cardiometabolic health and examine which measures of body composition best predict the risk of NCD.

## Materials and methods

The PRISMA (Preferred Reporting Items for Systematic review and Meta-Analysis) protocol was used for this systematic review^(27)^.

### Inclusion/exclusion criteria

Inclusion criteria were based on PICOS outline:

- Population: Subjects who had nutritional status (BMI or body composition) measured at baseline at any time from birth up to 5 years of age with a follow-up time ≥ 10 years.
- Intervention/exposure: Exposure to any of the following body composition measurements: SF, BIA, dual-energy X-ray absorptiometry (DXA/DEXA) scan, isotope dilution and PEA POD air displacement plethysmography. Despite only being proxy indicators of body composition, we also included BMI and Ponderal index (PI) (measures of weight relative to height).
- Comparator/control: Studies with and without a control group are included.
- Outcome: Cardiometabolic NCD (coronary artery disease, type 2 diabetes, metabolic syndrome (MetS)) and their associated risk factors (obesity, blood pressure (BP), blood glucose levels, lipid levels, WC) were measured ≥ 10 years after exposure.
- Study design: All study designs were considered eligible.

The review excluded studies with a high-risk study population, grey literature, unpublished studies, reviews, non-human studies and studies not published in English, in full format and before 1990.

### Search strategy

The search was completed independently by two authors in three databases: *Embase Classic +* *Embase*, *Ovid MEDLINE (R) and In-Process & Other non-Indexed Citations and Daily*, and *Global Health*. The final search was conducted on 27 July 2020. A detailed search strategy is shown in Appendix A.

### Study selection

All records generated from the search were imported into Mendeley Reference Manager (version 1·19·4) and were screened by title and abstract. Articles that were deemed relevant or where more information was needed to determine relevance were screened by full text.

### Data extraction

A data extraction form developed for this review was used to extract information from eligible studies. When obtainable, the following information was extracted: author, year, title, country, study design, sample size, percentage female, inclusion and exclusion criteria, type of exposure and assessment method, type of outcome and assessment method, years of follow-up, adjustment for current body size, key findings and strength of evidence (a judgement made by us based on numerous factors including study type, quality/risk of bias and certainty of results).

### Data analysis

Due to heterogeneity amongst studies identified, the analysis is presented as a narrative synthesis. Results from high-income countries and low- and middle-income countries are analysed separately and should not be compared.

### Assessing risk of bias

An individual risk of bias assessment for each study was determined using the ‘Quality appraisal checklist for quantitative studies reporting correlation and associations’ in ‘Methods for the development of NICE public health guidance’^(28)^.

### Study protocol

A pre-registered protocol for this review can be found at: https://www.crd.york.ac.uk/prospero/display_record.php?RecordID = 188 393

## Results

### Study selection

**Fig. 1:**
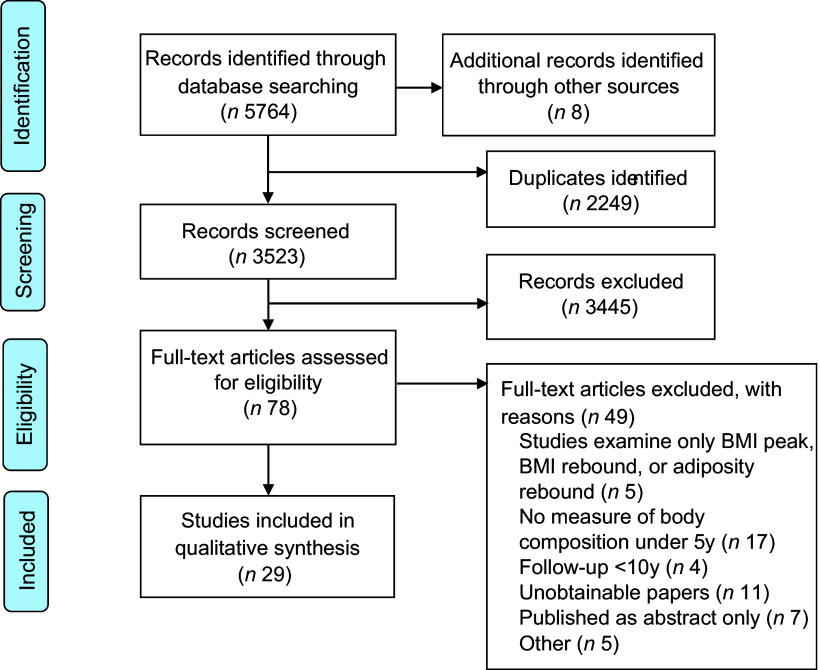
Study selection flow diagram

Selection process and search results are presented in Fig. 1. The search generated 5772 records. Following deduplication and initial screening of titles and abstract, seventy-eight articles were eligible for full-text review. Of these, forty-nine did not meet the inclusion criteria which led to a total of twenty-nine studies included in the review.

### Study characteristics

Study characteristics are presented in Table 1. Most studies were from high-income countries (*n* 21, 72 %), and all but one study used BMI as the indicator of early life exposure/body composition (*n* 28, 97 %). Few studies (*n* 7, 25 %) used an additional indictor, which was either PI (*n* 4, 40 %) or SF thickness (*n* 3, 38 %). No studies used direct measures of body composition: BIA, (DXA/DEXA) scan, isotope dilution and PEA POD air displacement plethysmography.

**Table 1:** Study characteristics of included studies

Author (year)	Country	Study Design	Sample size	Type of Exposure	Age at Outcome Assessment	Mean age in years	Outcome Assessed	Quality of study External | Internal validity validity
High-income countries								
Angbratt (2011)^(31)^	Sweden	Retrospective cohort	3579	BMI, PI	∼15	14·5	Obesity (BMI > 30)	+ ++
Barker (2005)^(32)^	Finland	Birth cohort 1934–44	444 (clinical subset *n* 2003)	BMI, PI	∼37–64	n/r	Coronary risk factors	+ ++
East (2020)^(33)^	Chile	Retrospective cohort (Santiago Longitudinal Study)	1000	BMI	∼23	n/r	Cardiometabolic risk	++ +
Eriksson (2003)^(34)^	Finland	Birth cohort 1934–44	444 (clinical subset *n* 2003)	BMI, PI	∼56–66 (n/r)	n/r	Obesity (BMI > 30)	+ +
Eriksson (2015)^(35)^	Finland	Birth cohort 1934–44	13345 (clinical subset *n* 2003)	BMI	40+	Clinical subsample: 62	T2D	++ ++
Evensen (2019)^(36)^	Norway	Prospective cohort (The Tromsø Study: Fit Futures)	907	BMI	∼15–20	n/r	Overweight/obesity (BMI > 25)	++ ++
Geserick (2018)^(37)^	Germany	Retrospective cohort	34196	BMI	∼18	n/r	Obesity (BMI > 30)	++ +
Giudici (2017)^(38)^	France	Retrospective cohort	1919	BMI	∼20–60	30·7	MetS risk factors, WC	− +
Golab (2018)^(39)^	Netherlands	Birth cohort (Generation R Study)	593	Skinfold thickness	∼10	n/r	Adiposity	− +
Graversen (2014)^(40)^	Finland	Birth cohort 1996	2120	BMI	∼31	n/r	BMI, WC, MetS risk factors	++ ++
Holland (1993)^(41)^	Great Britain	Birth cohort (NHSD)	2830	BMI	∼36	n/r	BMI, BP	− ++
Howe (2010)^(42)^	UK	Birth cohort (Avon)	5113	BMI, PI	∼15	15·5	CVD risk	− ++
Howe (2014)^(43)^	England	Birth cohort (Avon)	3154	BMI	∼17	17·8	BP	− ++
Huang (2012)^(44)^	Australia	Birth cohort (Raine)	1053	BMI, skinfold thickness	∼17	n/r	CVD risk	++ +
Johnson (2014)^(45)^	Great Britain	Birth cohort (NSHD)	1273	BMI	∼60–64	n/r	cIMT	++ ++
Johnson (2017)^(46)^	USA	Birth cohort (Fels Longitudinal Study)	350	BMI	∼20–60	n/r	Adiposity	++ ++
Kwon (2017)^(47)^	USA	Birth cohort (Iowa Fluoride Study)	1093 (clinical subset at 8 years *n* 495, 19 years *n* 314)	BMI	∼8, 19	n/r	Adiposity	+ +
Lagström (2008)^(48)^	Finland	Prospective randomised trial (STRIP)	541	BMI	∼13	n/r	Obesity (BMI > 30)	++ +
Lammi (2009)^(49)^	Finland	Case-control	128	BMI	∼15–39	34·5	T2D diagnose	− +
Salonen (2009)^(29)^	Finland	Birth cohort 1934–44	588 (clinical subset: 2003)	BMI	∼56–70	61·5	BMI, MetS risk factors	+ ++
Skidmore (2007)^(50)^	Great Britain	Birth cohort (NSHD)	2311	BMI	∼53	n/r	Lipid levels	+ ++
Ziyab (2015)^(51)^	UK	Birth cohort (The Isle of Wight birth cohort)	1240	BMI	∼18	n/r	BP	++ ++
Low- and middle-income countries								
Bhargava (2004)^(52)^	India	Birth cohort (New Deli Birth Cohort)	1526	BMI	∼18	n/r	BP, adiposity	− +
Corvalán (2007)^(30)^	Guatemala	Prospective cohort (INCAP study)	710	BMI	∼33–35	32·7	Adiposity	+ ++
Fall (2008)^(53)^	India	Birth cohort (New Deli Birth Cohort)	1583	BMI	∼26–32	n/r	Adiposity, MetS risk factors	− +
Krishnaveni (2015)^(54)^	India	Birth cohort (Mysore Parthenon)	414	BMI, skinfold thickness	∼13·5	n/r	Adiposity, T2D risk factors	++ ++
Raghupathy (2010)^(55)^	India	Birth cohort (Vellore 1969–73)	2218	BMI	∼26–32	28·3	T2D risk factors	+ ++
Sachdev (2005)^(56)^	India	Birth cohort (New Deli Birth Cohort)	1526	BMI	∼26–32	n/r	Adiposity	− +
Weitz (2014)^(57)^	Solomon Islands	Prospective cohort	540	BMI, skinfold thickness	∼19–38	n/r	TC, TG	++ ++

BMI: body mass index; BP: blood pressure; cIMT: carotid intima-media thickness; MetS: metabolic syndrome; n/r: not reported; TC: total cholesterol; TG: total glucose; T2D: type 2 diabetes; PI: Ponderal index; WC: waist circumference.

(++) means ‘good quality of study’, (+) means ‘adequate quality of study’, (-) means ‘poor quality of study’.

Sample size included in the analysis ranged from 128 to 34 196 participants. Participants were most often drawn from existing cohorts (of which nineteen were birth cohorts), and four studies from high-income countries recruited the study population from health care registers from the respective countries. All studies were representative cohorts, although the study population in one Finnish study were exclusively normal weight in adulthood^(29)^ and the Guatemalan study population had a high prevalence of stunting (53 % stunted by age 7)^(30)^.

Studies included in the review were a mix of good, adequate and poor quality. External validity for nine studies was rated to be of poor quality due to various reasons, e.g. reduced power and significant proven differences between study population and participants who were lost to follow-up.

### Synthesis of results

Tables 2–5 present a summary of included studies reporting on CVD outcomes, glucose metablisme, MetS outcomes and obesity-related outcomes respectively. A detailed summary of all studies can be found in Appendix B. The following section describes the results of the included studies.

**Table 2:** Summary of studies reporting on CVD outcomes

Author (year)	Sample size	Type of exposure	Age at outcome assessment	Mean age in years	CVD outcomes
High-income countries					
Barker (2005)^(32)^	444 (clinical subset *n* 2003)	BMI, PI	∼37–64	n/r	↑ BP with a 1SD z-score increase in BMI (7 years, 10 years). Associations stronger in males. Females: ↑ BP in subjects with low BMI at birth ↓ BMI (6 months, 2 years) associated with coronary events. ↑ BMI (4 years onwards) associated with coronary events. BMI (2 years, 11 years) predicted coronary events in a simultaneous regression. The hazard ratios associated with an increase in BMI of 1 sd were 0·62 at 2 years of age and 1·35 at 11 years. Males: ↑ brachial sBP in subjects with low BMI at birth ↓BMI and PI at birth and BMI (1 years, 2 years) predicted coronary events. ↑ BMI (2 years onwards) associated with coronary events. The hazard ratios associated with an increase in BMI of 1 sd were 0·76 at 2 years of age and 1·14 at 11 years.
Holland (1993)^(41)^	2830	BMI	∼36	n/r	Females: ↑ BP with 1 unit increase in BMI SDS (birth to 7 years) Males: ↑ BP with 1 unit increase in BMI SDS (birth to 7 years)
Howe (2010)^(42)^	5113	BMI, PI	∼15	15·5	↑ CVD risk with 1SD BMI z-score increase (5–5·5 years, 7–8·5 years, 8·5–10 years)
Howe (2014)^(43)^	3154	BMI	∼17	17·8	↑ BP with a 1SD z-score increase in BMI (7 years, 10 years). Associations stronger in males. No association between BMI < 7 years and later BP. Highest BP was seen in those with a low birthweight as well as overweight or obesity at age 2 and age 17. Females: ↑ sBP and dBP in subjects with low BMI at birth Males: ↑ sBP in subjects with low BMI at birth
Huang (2012)^(44)^	1053	BMI, skinfold thickness	∼17	n/r	BP, insulin, glucose, TG, BMI, TC, HDL-C, LDL-C and high-sensitive CRP were assessed, and study population was divided into ‘high-risk’ and ‘low-risk’ cluster according to their results. Trajectories of BMI and SF were then analysed separately for each group. All findings are in high risk *v*. low risk cluster. Females: ↑ BMI (1–5 years) ↑ SF thicknesses(1–5 years) Males: ↑ BMI (3 years) ↑ SF thicknesses(3–5 years)
Johnson (2014)^(45)^	1273	BMI	∼60–64	n/r	Males: ↑ Odds of high cIMT with 1 unit increase in z-score BMI (4 years, 20 years) in the upper *v*. the 3 lower quartiles. Females: no association.
Skidmore (2007)^(50)^	2311	BMI	∼53	n/r	↓HDL-C with 1 unit increase in BMI SDS (7–15 years). ↓TC with 1 unit increase in BMI SDS (2 and 4 years)
Ziyab (2015)^(51)^	1240	BMI	∼18	n/r	Trajectories of BMI were analysed, study population was divided into ‘early persistent obesity’, ‘delayed overweight’ and ‘normal’. ↑ BP in early persistent obesity trajectory and delayed overweight *v*. normal
Low- and middle- income countries					
Weitz (2014)^(57)^	540	BMI, skinfold thickness	∼19–38	n/r	Females: ↑ TC associated with BMI Z residuals (6–11 years and 12–19 years) ↑ TC associated with subscapular SF residuals (0–5 years) ↑ TG associated with BMI Z residuals (12–19) Males: ↑ TC associated with BMI Z residuals (0–5 years) ↑ TG associated with subscapular SF thickness (6–11 years and 12–19 years)

BP: blood pressure; cIMT: carotid intima-media thickness; dBP: diastolic blood pressure; HDL-C: HDL-cholesterol; HR: hazard ratio; n: sample size included in analysis; n/r: not reported; PI: Ponderal index; sBP: systolic blood pressure; SDS: standard deviation score; SF: skinfold; TC: total cholesterol; TG: total glucose.

↑ = increased;↓ = decreased.

**Table 3:** Summary of studies reporting on glucose metabolism outcomes

Author (year)	Sample size	Type of Exposure	Age at Outcome Assessment	Mean age in years	Glucose metabolism outcomes
High-income countries					
Barker (2005)^(32)^	444 (clinical subset *n* 2003)	PI, BMI	∼34–64		For both sexes fasting plasma glucose and insulin fell both with increasing birth weight and with increasing BMI at 2 years. ↑ plasma glucose and insulin resistance associated with low birth weight (LBW), low BMI at 2 years and an increase in sd scores for BMI from age 2 to 11.
East^(33)^	1000	BMI	∼23		Hyperglycaemia was assessed, and study population was divided into ‘risk present’ and ‘risk absent’ cluster according to their results. Trajectories of BMI were then analysed separately for each group. ↑ BMI (2–23 years) in subjects with hyperglycaemia Results are presented as ‘risk present *v*. risk absent’: slope 6 months–5 years: 0·05 *v.* –0·12, intercept 5 years: 18·05 *v.* 16·78, slope 5–2 years: 3·17 *v.* 2·93
Eriksson (2015)^(35)^	13 345 (clinical subset *n* 2003)	BMI	40+	Clinical subsample: 62	Outcomes were assessed, study population was divided into ‘BMI above the median at 11 years’ (group 1) and ‘BMI below the median at 11 years’ (group 2). Trajectories of BMI were then analysed separately for each group. ↓Risk of T2D with each unit increase in BMI z-score (0–7 years) (group 1). ↑ Risk of T2D with each unit increase in BMI z-score (7 years onwards) (group 1). ↓Risk of T2D with each unit increase in BMI z-score (0–11 years) (group 2). In group 1, low BMI at birth and at 2 years and high BMI at 11 years was associated with T2D. In group 2, low BMI was associated with T2D.
Lammi (2009)^(49)^	128	BMI	∼15–39	34·5	↑ Risk of T2D with a gain of 1 kg/m^2^ in the minimum BMI (3–11 years) *v.* controls
Low- and middle- income countries					
Bhargava (2004)^(52)^	1526	BMI	∼18	n/r	Low PI at birth associated with increased plasma glucose and insulin concentrations and insulin resistance at follow-up. It was not associated with IGT/DM. ↑ Odds of IGT/DM with low BMI at 1 years. ↑ Odds of IGT/DM with 1SD BMI z-score increase (2–12 years).
Fall (2008)^(53)^	1583	BMI	∼26–32	n/r	↑ Odds of T2D per sd score BMI increase (2–11 years)
Krishnaveni (2015)^(54)^	414	BMI, skinfold thickness	∼13·5	n/r	↑ Fasting insulin concentrations and HOMA-IR associated with fat gain (5 years onwards)
Raghupathy (2010)^(55)^	2218	BMI	∼26–32	28·3	↓Odds of IGT/DM with a PI at birth close to a Z-score of 0 ↓Odds of IGT/DM with a childhood BMI close to a Z-score of 0 ↑ Odds of IGT/DM with increase in BMI z-scores from birth-adulthood, infancy-adulthood, childhood-adulthood, adolescent-adulthood. Association grew stronger with age.
Weitz (2014)^(57)^	540	BMI, skinfold thickness	∼19–38	n/r	Females: ↑ TG associated with BMI z residuals (12–19) Males: ↑ TG associated with subscapular SF thickness (6–11 years and 12–19 years)

DM: diabetes mellitus; HOMA-IR: Homeostatic Model Assesment for Insulin Resistance; IGT: impaired glucose tolerance; LBW: low birth weight; n: sample size included in analysis; n/r: not reported; SDS: standard deviation score; SF: skinfold; TG: total glucose; T2D: type 2 diabetes.

↑ = increased;↓ = decreased.

**Table 4:** Summary of studies reporting on metabolic syndrome outcomes

Author (year)	Sample size	Type of Exposure	Age at Outcome Assessment	Mean age in years	Metabolic syndrome outcomes
High-income countries					
East (2020)^(33)^	1000	BMI	∼23	n/r	MetS was assessed, and study population was divided into ‘risk present’ and ‘risk absent’ cluster according to their results. Trajectories of BMI were then analysed separately for each group. ↑ BMI (6 months–23 years) in subjects with MetS. Results are presented as ‘risk present *v*. risk absent’: slope 6 months–5 years: 0·07 *v.* –0·13, intercept 5 years: 18·44 *v.* 16·59, slope 5–23 years: 4·22 *v.* 3·03.
Giudici (2017)^(38)^	1919	BMI	∼20–60	30·7	MetS was assessed, the study population was divided into ‘with Mets’ and ‘without Mets’. Trajectories of BMI and WC were then analysed separately for each group. ↑ BMI (4–6 years, 7–10 years) in adults with MetS *v*. without MetS. ↑ _a_WC associated with higher BMI (0–10 years). No association between low _a_HDL-C and BMI in childhood. No association between hyperglycaemia and BMI in childhood. No association between _a_BP and BMI in childhood.
Graversen (2014)^(40)^	2120	BMI	∼31	n/r	↑ Risk of MetS with a BMI in the ≥ 50–< 75 and ≥ 95 percentile (3 years) *v*. those in the ≥ 5–< 50 percentile. ↑ Risk of MetS with a BMI at the ≥ 95 percentile (4 years, 5 years) *v*. those in the ≥ 5–< 50 percentile.
Salonen (2009)^(29)^	588 (clinical subset: 2003)	BMI	∼56–70	61·5	Males: ↓Odds of MetS with 1SD z-score increase in BMI (0–2 years) Females: No statistical association was seen in women.
Low- and middle-income countries					
Fall (2008)^(53)^	1583	BMI	∼26–32	n/r	↑ Odds of MetS per sd score BMI change (2–11 years). Subjects with MetS had higher mean BMI than the cohort mean at all ages from birth.

_a:_ adult; BP: blood pressure; HDL-C: HDL-cholesterol; MetS: metabolic syndrome n; sample size included in analysis; n/r: not reported; WC: waist circumference.

↑ = increased;↓ = decreased.

**Table 5:** Summary of studies reporting on obesity-related outcomes

Author (year)	Sample size	Type of Exposure	Age at Outcome Assessment	Mean age in years	Obesity-related outcomes
High-income countries					
Angbratt (2011)^(31)^	3579	BMI, PI	∼15	14·5	Moderate/small correlations was found between PI at birth, 1·5 years and BMI at 2·5 years with BMI at 15 years (*r* < 0·5). Strong correlation between BMI at 5 years, 7 years, 10 years with _a_BMI
Barker (2005)^(32)^	444 (clinical subset *n* 2003)	BMI, PI	∼37–64	n/r	Moderate/small correlations was found between PI at birth, 1·5 years and BMI at 2·5 years with BMI at 15 years (*r* < 0·5). Strong correlation between BMI at 5 years, 7 years, 10 years with _a_BMI
East (2020)^(33)^	1000	BMI	∼23	n/r	Abdominal obesity was assessed, and SP was divided into ‘risk present’ and ‘risk absent’ cluster according to their results. Trajectories of BMI were then analysed separately for each group. ↑ BMI (6 months–23 years) in subjects with abdominal obesity Results are presented as ‘risk present *v*. risk absent’: slope 6 months–5 years : 0·05 *v.* –0·17, intercept 5 years : 18·12 *v.* 16·25, slope 5–23 years : 4·20 *v.* 2·70
Eriksson (2003)^(34)^	444 (clinical subset *n* 2003)	BMI, PI	∼56–66	n/r	Obesity was assessed, and SP was divided into ‘obese’ and ‘non-obese’ cluster according to their results. Trajectories of BMI and PI were then analysed separately for each group. ↑ BMI (0–12) in obese adults *v*. non-obese Females: ↑ Prevalence of BMI > 30 in adults with a PI > 18·5 at birth *v*. those with a PI < 18·5 Males: ↑Prevalence of BMI > 30 in adults with a PI > 18·5 at birth *v*. those with a PI < 18·5
Evensen (2019)^(36)^	907	BMI	∼15–20	n/r	Females: ↑Odds of higher _a_FMI (by DXA) in overweight/obese children (2·5 years) *v*. normal weight ↑Odds of higher _a_WC in overweight/obese children (6 years) *v*. normal weight Males: ↑Odds of higher _a_WC and _a_FMI (by DXA) in overweight/obese children (6 years) *v*. normal weight
Geserick (2018)^(37)^	34 196	BMI	∼18	n/r	Accelerated annual change in BMI SDS (≥ 0·2 to < 2·0) in children 2–6 years associated with overweight/obesity at 18 years
Giudici (2017)^(38)^	1919	BMI	∼20–60	30·7	WC, sBP, dBP, total glucose, TG, HDL-C, LDL-C were assessed and SP was divided into ‘with Mets’ and ‘without Mets’ according to their results. Trajectories of BMI and WC were then analysed separately for each group. ↑ _a_WC associated with higher BMI (0–10 years)
Golab (2018)^(39)^	593	Skinfold thickness	∼10	n/r	In infancy SF thicknesses were used as a measure of adiposity whilst at age 10 FM was measured using a DXA scanner. ↑Central-to-total FM ratio (1·5 months) associated with higher BMI, FMI, subcutaneous FMI at 10 years ↑Total subcutaneous FM (6 months, 2 years) associated with higher BMI, FMI, subcutaneous FM at 10 y No association between exposure and visceral fat, pericardial fat and liver fat at 10 y
Graversen (2014)^(40)^	2120	BMI	∼31	n/r	Linear relationship with BMI (0–5 years) associated with _a_BMI and _a_WC
Johnson (2017)^(46)^	350	BMI	∼20–60	n/r	↑BMI (20 years) and DXA-measured FFMI (20 years, 30 years) with 1 unit increase in infant BMI Z-score No associations between infant BMI and DXA-measured FFMI after age 30
Kwon (2017)^(47)^	1093 (clinical subset at 8 years *n* 495, 19 years *n* 314)	BMI	∼8, 19	n/r	Trajectories of BMI were analysed, trends were identified, SP was divided into ‘consistently low BMI in childhood’ (group 1), ‘steep increase in BMI second year of life’ (group 2), ‘steep increase in BMI the first year of life’ (group 3), ‘consistently high BMI in childhood’ (group 4). FMI at age 8 and 19 were derived using DXA scans. Females: ↑FMI (8 years, 19 years) in group 3 and 4 *v*. group 1 and 2 Males: no statistical association.
Lagström (2008)^(48)^	541	BMI	∼13	n/r	Owerweight/obesity was assessed, SP was divided into ‘overweight/obese’ and ‘normal’. Trajectories of BMI were then analysed separately for each group. All results are in overweight/obese *v*. normal children. Females: ↑BMI (5 years onwards) associated with overweight/obesity Males: ↑BMI (8 years onwards) associated with overweight/obesity
Low- and middle-income countries					
Corvalán (2007)^(30)^	710	BMI	∼33–35	32·7	FFM and PBF were estimated using predictive equations from hydrostatic weight measurements in a similar population ↑ _a_BMI, FFM associated with BMI (at birth) ↑ _a_BMI, FFM, PBF, AC associated with BMI (0–1 years and 3–7 years) - changes in BMI between 3–7 years were more strongly associated with FM and AC in adulthood than with FFM No association between BMI at 1–3 years and outcomes
Krishnaveni (2015)^(54)^	414	BMI, skinfold thickness	∼13·5	n/r	At age 0–5 years adiposity was derived from SF and from 10 years onwards researchers used bioelectrical impedance analysis. ↑FM at birth and fat gain during infancy and childhood associated with BMI and SF at follow-up. ↑WHR and PBF associated with fat gain (0–6 years). ↑_a_SF thickness associated with greater lean tissue gain (2–5 years). ↑ _a_BMI associated with greater lean tissue gain (0–13·5 years).
Sachdev (2005)^(56)^	1526	BMI	∼26–32	n/r	Adiposity measures derived from SF ↑Childhood BMI correlated with _a_BMI (correlation strengthened with age) PI at birth associated with lean residual ↑BMI at birth associated with high _a_BMI and lean residual BMI in infancy and early childhood correlated more strongly with _a_FFM than with _a_FM ↑BMI in late childhood and adolescence associated with ↑ _a_FM

_a_: adult ; FM: fat mass; FFMI: fat-free mass index; FM: fat mass; FMI: fat mass index; n; sample size included in analysis; n/r: not reported; PI: Ponderal index; SF: skinfold; WC: waist circumference.

↑ = increased; ↓ = decreased.

### Cardiovascular outcomes (Table 2)

#### High-income countries

A study looking at CVD risk found that females who had increased BMI and SF thicknesses from ages 1 to 5 years had increased risk of CVD (BMI: *P* < 0·001; SF thicknesses: *P* < 0·05). High-risk males had increased BMI at 3 years (*P* < 0·001) and increased SF thicknesses from 3 to 5 years (*P* < 0·001)^(44)^. Another study reporting on CVD risk did not find any association before 5 years, but reported that increased BMI in later childhood was associated with increased CVD risk^(42)^.

Four studies reported on BP. One found that low BMI at birth was associated with increased BP. The researchers also found that there was no association between BMI below 7 years and later BP; however, those subjects with the highest BP had a low birthweight and were overweight or obese at age 2 and time of exposure^(43)^. Another reported that a one unit increase in BMI standard deviation score (SDS) from birth to 7 years was associated with elevated BP. Changes in systolic blood pressure were greater in females than in males (1·4 mmHg *v*. 0·7 mmHg), but in contrast diastolic blood pressure was greater in males than in females (1·0 mmHg *v*. 0·5 mmHg)^(41)^. Another study measuring BP reported that children who became obese early in life and who had a delayed overweight (overweight at 10 and 18 years) had higher BP at follow-up than those with a healthy weight in childhood (all *P* < 0·001)^(51)^. A Finnish study found that systolic blood pressure fell both with increasing birth weight and increasing BMI at 2 years. The researchers also reported on the prevalence of coronary events and found that adults who experienced coronary events were smaller than average at birth and had a BMI below average at age 2. After age 2 and 4 (for boys and girls, respectively), their BMI increased progressively. The authors concluded that ‘*The risk of coronary events was more strongly related to the tempo of childhood gain in BMI than to the BMI attained at any particular age*’^(32)^.

Results from a British cohort examined carotid intima-media thickness and grouped the study populations (male and female) into quartiles. Boys with a BMI in the upper quartiles had increased odds of high carotid intima-media thickness with 1 unit increase in z-score BMI at 4 years (OR1·26; *P* = 0·03) *v*. boys with a BMI in the three lower quartiles. They found no such association in girls (*P* > 0·05)^(45)^.

Another British cohort reported on lipid levels at age 53 and found that a 1 sd increase in BMI at ages 2 and 4 was associated with lower levels of total cholesterol (*P* = 0·007 and *P* = 0·003, respectively) and an increase in BMI from 7 to 15 years was associated with lower levels of HDL-cholesterol with the association being stronger and greater in females. The researchers adjusted for the current body size^(50)^.

#### Low- and middle-income countries

A study from India found that fat gain measured by SF from 5 years onwards was associated with elevated systolic blood pressure in adulthood^(54)^.

A study from Melanesia reporting on total cholesterol found that BMI z residuals from 0 to 5 years in males were associated with increased total cholesterol. In females, there was a positive association between SF residuals from 0 to 5 years and CVD, and BMI z residuals from 6 to 11 years and CVD^(57)^.

### Glucose metabolism outcomes (Table 3)

#### High-income countries

Two Finnish studies reported the risk of type 2 diabetes (T2D). One study found that a gain of 1 kg/m^2^ in subjects who had the minimum BMI from 3 to 11 years had an increased risk of T2D *v*. those who gained less than 1 kg/m^2^ (OR1·87; *P* = 0·04)^(49)^. Another study reported that children who had a BMI above the study population median at 11 years had a decreased risk of T2D with each unit increase in BMI z-score from 0–7 years. After 7 years, the same group had an increased risk of T2D with each unit increase in BMI z-score. Association was greater and stronger in females (females: OR1·35; *P* = 0·004, males: OR1·23; *P* = 0·01). Researchers found that within the same group, a low BMI at birth and at 2 years and a high BMI at 11 years were associated with T2D. The group with a BMI below the study population median at 11 years had a decreased risk of T2D with each unit increase in BMI z-score from 0 to 11 years. In this group, a low BMI at birth was associated with T2D^(35)^.

A different Finnish study assessed fasting plasma glucose and insulin resistance and found that low birth weight, low BMI at 2 years and an increase in sd scores for BMI from 2 to 11 years were associated with raised fasting plasma glucose and insulin resistance in adulthood^(32)^.

A study reporting on hyperglycaemia in Chile found that subjects with hyperglycaemia typically had an increased BMI from approximately 2 years onwards^(33)^.

#### Low- and middle-income countries

A study from India looking at impaired glucose tolerance/diabetes mellitus (IGT/DM) found that a PI at birth and BMI in childhood close to a z-score of 0 were protective against IGT/DM (OR0·80; *P* = 0·04 and OR0·77; *P* < 0·001, respectively). Greater changes in BMI z-score from birth to adulthood were associated with increased odds of IGT/DM that grew stronger by age (all *P* < 0·001, see Appendix B for all OR). Researchers concluded that those with IGT/DM in adulthood were typically LBW infants and that IGT/DM was associated with low BMI in childhood, followed by an accelerated BMI gain between birth, infancy, childhood or adolescence and adulthood^(55)^.

Another study from India reporting on DM found that there were increased odds of diabetes per sd score BMI increase from 2 to 11 years (OR1·25; *P* = 0·01) and that subjects with diabetes had more rapid weight/BMI gain throughout infancy, childhood and adolescence as well as a lower BMI in infancy^(53)^. A different study, using the study population from the same birth cohort, found a similar association: increased odds of IGT/DM with a low BMI at 1 year and with a 1SD BMI z-score increase from 2 to 12 years, which attenuated after adjustment for current body size (OR1·36; *P* < 0·001 and OR1·26; *P* = 0·004, respectively). Researchers also found an association with low PI at birth and increased plasma glucose and insulin concentrations and insulin resistance at follow-up, and noted that subjects who developed DM/IGT typically had a lower PI and BMI up to the age of 2^(52)^. A study reporting on insulin concentrations did not find an association with BMI and SF in the first 5 years post-natal but did associate fat gain measured by SF from 5 years onwards with increased fasting insulin concentrations and insulin resistance^(54)^.

One study reporting on total glucose did not find any association with BMI and SF in early life but did find that BMI residuals in females aged 12–19 years were associated with increased total glucose. In males, the researchers reported that SF residuals at ages 6–11 years and 12–19 years were associated with increased levels of total glucose^(57)^.

### Metabolic syndrome outcomes (Table 4)

#### High-income countries

Four studies reported on MetS. One study found no association with MetS and body size at birth and 2 years. However, changes in BMI in infancy were predictive, with a 1 sd z-score increase in BMI from 0 to 2 years in males associated with decreased odds of MetS in adulthood (OR0·53;0·33–0·87). Though there were similar observations in women, the changes were not statistically significant. Researchers adjusted for current body size and did not report unadjusted results^(29)^.

Another study reported that subjects with a BMI in the ≥ 50–< 75 and ≥ 95 percentile had increased risk of MetS *v*. those in the ≥ 5–< 50 percentile (RR1·9 *v*. RR1·6). Subjects with a BMI above the ≥ 95 percentile at 4 years and 5 years had a slightly greater risk of MetS *v*. those in the ≥ 5–< 50 percentile (RR2·5 *v*. RR2·4)^(40)^. Similarly, another study from France found that subjects with MetS had an increased BMI at 4–6 years and 7–10 years (*P* = 0·01 and *P* < 0·001, respectively)^(38)^.

A Chilean study reported that subjects who had MetS, had higher and faster growth in BMI from ages 6 months–23 years^(33)^.

#### Low- and middle-income countries

Fall *et al.* found that the odds of MetS increased per sd score BMI change from 2 to 11 years (OR1·48; *P* < 0·001) and that subjects with MetS had a more rapid weight/BMI gain throughout infancy, childhood and adolescence^(53)^.

### Obesity-related outcomes (Table 5)

#### High-income countries

Eleven studies reported on obesity-related outcomes. A study reporting on BMI did not find any association before the age of 5. BMI from 5 years onwards was associated with overweight/obesity in females whilst this association in males was seen from age 8 years onwards^(48)^.

Angbratt *et al.* found a small correlation with PI at birth, 1·5 years and BMI at 2·5 years (*r* < 0·5) and overweight/obesity at follow-up whilst BMI at age 5, 7 and 10 years was strongly correlated with BMI at follow-up (*r* > 0·5)^(31)^. Another study found that a linear relationship with BMI at ages 0–5 years was associated with higher BMI and WC at follow-up^(40)^.

One study reported that an increased BMI from 0 to 10 years was associated with elevated WC at follow-up (*P* < 0·001)^(38)^. Similarly, a study reported that obese subjects at follow-up had a high BMI between 0 and 12 years (all *P* < 0·001). The researchers also found that females and males with a PI < 18·5 at birth had increased odds of becoming obese adults *v*. those with a PI > 18·5 (OR3 and OR4, respectively)^(34)^. Similarly, another study found that subjects with abdominal obesity had an increased BMI gain from infancy to follow-up *v*. those without abdominal obesity^(33)^.

A study reporting on the accelerated annual change in BMI SDS found that an annual change of ≥ 0·2 to < 2·0 BMI SDS in children 2–6 years increased their risk of overweight/obesity later in life *v*. children with a stable BMI between age 2 and 6 years (RR1·43; CI 1·35, 1·49)^(37)^.

One study reported on infant BMI only and found that a 1 unit increase in infant BMI z-score was associated with high BMI at 20 years (*β* = 0·70; CI 0·31, 1·09; *P* < 0·001) and high DXA-measured fat-free mass index at 20 years (*β* = 0·75; CI 0·37, 1·12; *P* < 0·001) and 30 years (*β* = 0·34; CI 0·12, 0·56). They found no association between infant BMI and body composition after age 30^(46)^.

Golab *et al.* reported on different adiposity measures using SF and found that an increased central-to-total fat mass (FM) ratio at 1·5 months and increased total subcutaneous FM at 6 months and 2 years was associated with higher BMI and fat mass index at follow-up^(39)^. Similarly, another study reported that females who had a steep increase in BMI during the first year of life had higher DXA-measured fat mass index at follow-up. There was no association in males^(47)^.

The Norwegian study showed that females who were overweight/obese at 2·5 years had increased odds of a higher fat mass index measured by DXA at follow-up *v*. those with a normal weight at 2·5 years (OR: 2·00; *P* < 0·05); however, the association was stronger with overweight/obesity at 6 years and increased WC at follow-up *v*. normal weight at 6 years (OR: 4·79; *P* < 0·001). In males, there was no association between overweight/obesity at age 2·5 and obesity-related outcomes at follow-up; however, overweight/obesity at 6 years was associated with increased odds of increased WC (OR: 5·56; *P* < 0·001) and DXA-measured fat mass index (OR: 4·14; *P* < 0·001) at follow-up^(36)^.

#### Low- and middle-income countries

A Guatemalan study found an association between BMI at birth and BMI (*β* = 0·33; *P* < 0·05) and fat-free mass (FFM) (*β* = 0·49; *P* < 0·01) at follow-up. FFM was estimated using predictive equations from hydrostatic weight measurements in a similar population. Results also showed that BMI at 0–1 years and 3–7 years was associated with BMI, FFM, percentage body fat and abdominal circumference, and that changes in BMI from 3 to 7 years were most strongly associated with adult FM and abdominal circumference (see Appendix B for details). There was no association between BMI at ages 1–3 years and measured outcomes at follow-up^(30)^.

A similar study in India found a correlation between BMI in childhood and BMI at follow-up; the correlation strengthened with age (6 months: *r* = 0·19, 2 years: *r* = 0·24, 5 years: *r* = 0·32, 14 years: *r* = 0·65). The study reported that PI at birth was associated with FFM in adulthood and that BMI in infancy and early childhood correlated more strongly with adult FFM whilst increased BMI in late childhood and adolescence was associated with adult FM. FFM and FM were derived from SF^(56)^.

Another Indian study reported an association between fat gain from 0 to 6 years and waist–hip ratio, BMI and percentage body fat at follow-up. In addition, the results showed that greater lean tissue gain from 2 to 5 years and 0–13·5 years was associated with SF thickness and BMI at follow-up, respectively. FM, FFM and percentage body fat were derived from SF up to age 5 whereafter researchers used BIA^(54)^.

## Discussion

### Summary

The major finding from our review is that evidence on childhood body composition and later-life NCD is severely limited. Though four studies assessed SF thickness in childhood, we did not find any using the more direct and technically superior methods such as isotope dilution, plethysmography or DXA. We did however find numerous studies using BMI (and a smaller number using PI)—but it is important to note that these are only proxy measures of body composition. Among children of the same age, sex and BMI, the level of body fat may vary twofold^(58)^. Even with BMI as the childhood exposure variable, associations with later NCD are difficult to interpret due to marked inter-study heterogeneity, especially in terms of NCD measure and age at follow-up. Varied approaches to analysing, reporting and presenting data in addition to disparities of cut-off points add to the challenge of interpreting what is. Most studies showed that childhood BMI is associated with later-life cardiometabolic NCD risk and that changes in BMI rather than absolute BMI appear to be important. Some studies also showed sex-specific differences. Most studies were unadjusted for current body size and thus the independent effect of childhood BMI is open to question. Because most studies were from high-income settings, wider generalisability to populations in low- and middle-income settings is unknown.

### Interpretation of findings

Most of our interpretable data uses BMI as the childhood exposure variable. BMI is widely used to categorise nutritional status because it is simple and can be compared with reference standards^(59)^. Many people and even many non-specialist scientists/clinicians also view it as ‘*an indicator of body fatness*’^(60)^ – hence why it was so common in our search results. It is however just an indicator of variability in weight relative to height, not variability in FM and it cannot differentiate between FM and FFM. In children, this issue is further complicated by the variety of other factors such as age, sex, pubertal status and ethnicity. In relation to using BMI as an indicator of body fat in early life, another big limitation arises: specifically, that low BMI at birth and during infancy can act as a proxy for low FFM^(8)^, and hence as a marker of poor capacity for metabolic homeostasis^(61)^. This is highly problematic as this implies that greater relative weight may index different components of body composition at different time points. Although several studies in this review have shown that infant weight gain is protective of NCD in later life and that both low BMI at birth and in infancy *and* high childhood BMI are associated with an increased risk of NCD, the lack of information about the relationship between BMI and body composition makes it difficult to interpret the data and establish clear associations. Infancy is a particularly challenging period to investigate, as low BMI may indicate low FFM, whereas rapid BMI increases over time may indicate fat deposition through catch-up growth^(61)^.

In the studies that did differentiate between FM and FFM, low BMI in infancy and high BMI in childhood both predicted later NCD risk. This pattern links with the ‘capacity-load model’ which hypothesises that increased size at birth indicates a greater metabolic (homoeostatic) capacity, although those born in the highest weight categories may deviate from this pattern since a higher proportion of their weight is likely due to adipose tissue, which imposes a metabolic load^(19,62)^.

Several studies have associated birth weight and BMI in infancy with adult FFM, whilst BMI in later childhood was associated with both FM and FFM^(63–65)^. This is consistent with the studies included in this review. Four studies found that early BMI was associated with adult FFM whilst BMI in later childhood was associated with FM and FFM^(30,36,66,67)^. These were also the only studies distinguishing between FM and FFM while the rest of the studies reported on BMI as a whole. As BMI does not distinguish between FM and FFM, associations between early BMI and overweight/obesity are likely to be confounded by the gain in FFM, thus threatening the validity of its use. This might also explain why some studies did find an association between BMI and NCD, while others did not.

Other inconsistencies of our results among studies reporting on the same outcome measure can be explained by the follow-up time of the respective studies. In our review, years of follow-up varied widely from 10 to 70 years. In studies with relatively short follow-up time that show no association between exposure and outcome, it is likely that some subjects will go on to develop a NCD with time as most NCD do not develop until later in life^(68–70)^. T2D, for instance, is most commonly seen in people over the age of 45^(70)^, and only one-third studies in current review with T2D as an outcome had a study population above age 45^(35)^.

Lack of adjusting for current body size also impacts the interpretation of our findings. In 1999, Lucas *et al.* criticised researchers’ lack of understanding and communication of the statistical implications of this. Over 20 years later, our review suggests that the problem remains. Adjustment for current size is important because it implies that change in size as well as initial size can contribute to an association^(71)^. Previous reviews found that studies which had adjusted for current body size experienced a partial attenuation in effect size^(72)^ and that some associations completely disappeared after adjustment for current body size^(73)^. These discoveries show that studies that fail to undertake these adjustments may be confounded by adult body size, and therefore the observed associations might in fact reflect the tracking of childhood BMI across the lifespan instead of an actual association^(74)^.

In our review, eleven of twenty-nine studies adjusted for current body size^(29,32,42,43,46,50–53,55,75)^ and like previously reported, some associations attenuated or became statistically insignificant after adjustments or even reversed. However, while the researchers did comment on the effect adjustment for body size had on the results, most of the studies did not report both adjusted *v*. unadjusted results with respect to adjustment for current body size, making readers unable to analyse and interpret raw data to draw their own conclusions.

### Research in context

Similar to our review, Park *et al.* found an association between childhood overweight (2–12 years), unadjusted for adult body size and CVD outcomes in adulthood. They were unable to conclude that childhood overweight is an independent risk factor of adult CVD as the few studies that did report adjusted results were inconclusive. Furthermore, studies were mainly from high-income settings and thus the generalisability is limited^(25)^. In contrast, a review from Owen *et al.* in 2009 concluded that BMI gain from age 2 to 6 years had a weak inverse association (RR0·94, 95 % CI: 0·82, 1·07) with CHD risk^(76)^; however, statistical findings are weak with CI including 1·00. It is also important to notice that this conclusion was based on only three estimates and that the researchers for this review did not exclude cohorts with high-risk subjects (e.g. LBW babies). Owen *et al.* also reported that the inverse association between childhood BMI and CHD risk became weakly positive after age 7 years and grew stronger with age^(76)^. The inverse association is consistent with some studies included in the current review that found an association between low BMI in infancy and NCD risk factors in adulthood. The evidence supports the capacity-load model hypothesis^(77)^, where LBW means lower capacity, but an excessively high birthweight indicates macrosomia and also means lower homeostatic capacity in terms of ability to prevent NCD. Results from a number of studies in the current review suggest the same trend but whether this is due to the uncontrolled adjustments for current body size remains unanswered.

A review by Simmonds *et al.* based on high-income countries studies reported that BMI has poor sensitivity in identifying healthy-weight children, who later would become obese adults. However, BMI was found to be a reasonable accurate measure of obesity and thus can identify obese children who most likely will become obese adults. The researchers also reported that obese children had more than five times the risk of becoming obese adults than non-obese children (RR5·21; 95 % CI 4·50, 6·02)^(22)^. These findings are consistent with studies we found, which suggest a pattern whereby increased BMI at different ages throughout childhood is associated with NCD/obesity-related outcomes in adult life. However, a recent evaluation of a large dataset on children’s body composition found that below 6 years, there was a very weak relationship between high BMI and high body fatness, suggesting that the use of high BMI centile to index excess adiposity in young children is methodologically flawed^(77)^. Consistent with that, Simmonds *et al.* found that BMI was a poor predictor of obesity-related diseases, as only 40 % of adult diabetes and 20 % of CHD would occur in overweight/obese children^(22)^. This further underlines the importance of using better body composition measurements in future studies to examine the effect of childhood FM and FFM on adult NCD.

These three reviews also experienced challenges with the diversity in reporting, which for Simmonds *et al.* meant that a number of assumptions were made to conduct the meta-analysis and thus the reliability of the pooled estimate may be limited^(22)^. Due to the limitations of these reviews, results should be interpreted as a general trend rather than a precise estimate of an association or predictive accuracy.

Finally, a 2021 review focussed on NCD risk in survivors of childhood *under*nutrition/famine^(16)^. Though the exposure was to undernutrition (as assessed by standard anthropometric measures) and thus the opposite type of malnutrition to most studies in this review, authors also found an association with numerous NCD-related outcomes. Strength and consistency of the association also varied according to the outcome. Interpreting the reviews together, it seems that extremes at both ends of the malnutrition spectrum risk long-term adverse outcomes. Our observation that rate of weight change can mediate risk might offer insights into the mechanism spanning the two types of malnutrition. As that review highlights in the conclusions, this work on mechanisms is urgently needed.

### Limitations

All included studies controlled for some known confounders. However, all studies were also of observational design, and there is therefore an inherent risk of residual confounding affecting the results. Whilst it is impossible to control for all confounders, the most evident and important confounders should be taken into consideration. For example, Bhargava *et al.* did not adjust for socio-economic status (SES)^(52)^. SES is a well-known confounder and lack of controlling thereof may lead to significantly affected and incorrect effect size^(78)^. Though not simple, it might also have been possible to control for different times of follow-up, e.g. using age-standardised reporting of NCD-related outcome measures. Another important confounder is *in utero* growth and nutrition, as manifested by low birth weight and weight-for-gestational age. We hope that future studies will better take this into account and adjust accordingly since its impact on metabolic programming is well established. It is currently difficult to disentangle the relative contribution of *in utero* exposures from early child (u5 years) exposures on future NCD-related risk.

As mentioned, very few studies reported on actual body composition in relation to NCD which consequently highly limits our understanding of how FM and FFM in early childhood relates to later NCD risk. Alongside the problem of using BMI rather than other true measures of body composition, adjustment for current body size was a major limitation in this review. Since under half of the studies included in the narrative synthesis adjusted for current size, we are unable to confirm the independent effect of early childhood body size on long-term cardiometabolic health, and thus there is a possibility that the associations seen in studies that failed to undertake these adjustments is mediated through adult body size.

Several studies were greatly affected by the loss to follow-up and only four had an attrition rate below 20 %^(30,36,51,54)^. Seven studies reported a loss to follow-up above 60 % of the original cohort^(42–44,50,55,57,67)^, while eight did not address the attrition rate at all nor did they report it^(31,32,34,35,47–49,75)^.

None of the studies presented power calculations for their sample size, and only four studies^(33,43,49,55)^ identified reduced power as a limitation of their study and possible explanation for the lack of weak association/difference in groups.

### Research recommendations

This review has highlighted several areas needing urgent research attention.

Heterogeneity among future studies might be reduced by researchers reading our review when planning their own work and choosing outcome variables/measurement timings which can then be more directly compared with this past work. Checklists of key items to report in such nutrition/NCD follow-up studies might also help, forming the basis for a STROBE checklist extension^(79)^.

Based on the risk of bias assessment, it is recommended that future longitudinal studies improve their reporting on several potential sources of bias and include a flow diagram to demonstrate their participation and response rates. In particular, follow-up rates should be reported as well as implications should be clearly discussed.

BMI is a poor measure of adiposity as it does not distinguish between FM and FFM. Future work should use additional, more direct measures of adiposity, e.g. peapod, isotope dilution and DXA. These studies are urgently needed and could offer valuable insights into mechanisms linking early-life malnutrition (both undernutrition and overweight/obesity) with later-life NCD risk. Studies are also needed to explore the relative utility of different methods, e.g. which field-appropriate measures (such as BIA) are most closely associated with the more complex, costly but arguably more ‘gold standards’ measures such as DXA scans. Different tools are appropriate for different settings and different study types and budgets (e.g. large-scale population research might use field-friendly BIA machines which are portable and increasingly affordable; smaller studies requiring fewer individuals who can travel to a clinic setting might use a more robust but less portable measure like densitometry or DXA. Isotope dilution studies represent an intermediate option, accurate and viable for large field studies but relatively expensive for lab analyses).

Future research should also explore the impact of body composition at other stages of childhood and adolescence. Different ages may be more or less important in influencing the risk of later-life adult NCD. Our focus was on children aged under 5 years since these are the focus of much global policy and practice on child nutrition—but other ages also matter. What happens later on may either exacerbate or attenuate any effect of ‘adverse’ body composition in younger children. This would be important for programmers and policy-makers working on under 5’s to know.

Sex differences also matter and should be explored in future work. Not enough papers presented disaggregated data for us to comment on sex-specific differences in this review but differences are well recognised for the risks of both early-life malnutrition^(80,81)^ and adult NCD^(82)^. Thus, we hope that future researchers will carefully account for sex when documenting any links between early body composition and later-life NCD.

Finally, less than half of the studies in this review adjusted for current body size. Future studies should present both crude and adjusted associations.

## Conclusion

Our review found that early life (first 5 years of postnatal life) nutritional status, mostly as assessed by low BMI in infancy and increased BMI in later childhood, was often associated with increased risk of cardiometabolic diseases and risk factors in adult life. Although exact patterns of association varied in different studies and settings (i.e. whether absolute BMI or BMI change in childhood matter most), some evidence in our review suggests a pattern where low BMI at birth and infancy followed by a rapid weight gain in childhood exceeding recommended levels increases the risk of NCD. Whether different patterns of body composition mediate or explain some of these variations is not possible to say. Neither is it known whether childhood BMI is an independent risk factor for NCD in adulthood, or whether the association is simply mediated through adult overweight/obesity. Due to the limited evidence on nutritional status measures other than BMI, it is not possible to identify which measure of body composition best predicts NCD in adulthood.

We highlight several gaps in the literature: high-quality evidence on this topic—in particular, evidence from low- and middle-income countries and the use of more direct measures of body composition to better describe nutritional status. As technology is rapidly improving, better equipment/solutions are more accessible and can provide research with adequate measures of body composition. Findings from our review underline the necessity to improve and continue the tracking of body composition from birth to adulthood to help understand relevant mechanisms linking child nutrition to adult health/NCD. This has a key role to play in preventing the increasing rates of overweight/obesity among children and adults and ultimately prevent the rising prevalence of NCD.

## Appendix A

### Full search strategy

Database: Ovid MEDLINE(R) and In-Process & Other Non-Indexed Citations and Daily <1946 to July 27, 2020>

Search Strategy:

1. (diabetes type 2 or type 2 diabetes or diabetes mellitus or insulin resistance syndrome* or insulin resistan* or hyperglycaemia or hypertension or arteriosclerosis or cardiovascular disease* or cardio vascular disease* or blood pressure or coronary heart disease* or metabolic syndrome* or dysmetabolic syndrome* or cardio metabolic disorder* or cardiometabolic disorder* or lipid profile or lipid metabolism or lipid* profile or glucose metaboli* disorder* or glucose intoleran* or glucose toleran* or obes* or artheroscleros*).mp. (1781365)
2. ((infan* or baby or babies or child* preschool or preschool child or early childhood or young children or kindergarten* or children under 5 or children under five or under 5's) adj10 (body composition or body fat or fat percentage or fat % or lean mass or fat mass of fat free mass or muscle mass or grip strength or hand strength or antropometr* or skinfold or skinfold thickness or body mass index or BMI or percent fat or fat percent or densitometry)).mp. (5242)
3. 1 and 2 (2221)
4. ((infan* or baby or babies or child* preschool or preschool child or early childhood or young children or kindergarten* or children under 5 or children under five or under 5's) adj10 (body composition or body fat or fat percentage or fat % or lean mass or fat mass of fat free mass or muscle mass or grip strength or hand strength or antropometr* or skinfold or skinfold thickness or body mass index or BMI)).mp. [mp=title, abstract, original title, name of substance word, subject heading word, floating sub-heading word, keyword heading word, organism supplementary concept word, protocol supplementary concept word, rare disease supplementary concept word, unique identifier, synonyms] (5194)
5. 1 and 4 (2219)
6. body composition.mp. [mp=title, abstract, original title, name of substance word, subject heading word, floating sub-heading word, keyword heading word, organism supplementary concept word, protocol supplementary concept word, rare disease supplementary concept word, unique identifier, synonyms] (57816)
7. body fat.mp. [mp=title, abstract, original title, name of substance word, subject heading word, floating sub-heading word, keyword heading word, organism supplementary concept word, protocol supplementary concept word, rare disease supplementary concept word, unique identifier, synonyms] (33004)
8. (fat percentage or fat %).mp. [mp=title, abstract, original title, name of substance word, subject heading word, floating sub-heading word, keyword heading word, organism supplementary concept word, protocol supplementary concept word, rare disease supplementary concept word, unique identifier, synonyms] (271026)
9. fat mass.mp. [mp=title, abstract, original title, name of substance word, subject heading word, floating sub-heading word, keyword heading word, organism supplementary concept word, protocol supplementary concept word, rare disease supplementary concept word, unique identifier, synonyms] (20698)
10. lean mass.mp. [mp=title, abstract, original title, name of substance word, subject heading word, floating sub-heading word, keyword heading word, organism supplementary concept word, protocol supplementary concept word, rare disease supplementary concept word, unique identifier, synonyms] (5400)
11. fat free mass.mp. [mp=title, abstract, original title, name of substance word, subject heading word, floating sub-heading word, keyword heading word, organism supplementary concept word, protocol supplementary concept word, rare disease supplementary concept word, unique identifier, synonyms] (7537)
12. muscle mass.mp. [mp=title, abstract, original title, name of substance word, subject heading word, floating sub-heading word, keyword heading word, organism supplementary concept word, protocol supplementary concept word, rare disease supplementary concept word, unique identifier, synonyms] (17698)
13. grip strength.mp. [mp=title, abstract, original title, name of substance word, subject heading word, floating sub-heading word, keyword heading word, organism supplementary concept word, protocol supplementary concept word, rare disease supplementary concept word, unique identifier, synonyms] (11357)
14. hand strength.mp. [mp=title, abstract, original title, name of substance word, subject heading word, floating sub-heading word, keyword heading word, organism supplementary concept word, protocol supplementary concept word, rare disease supplementary concept word, unique identifier, synonyms] (15132)
15. anthropometr*.mp. [mp=title, abstract, original title, name of substance word, subject heading word, floating sub-heading word, keyword heading word, organism supplementary concept word, protocol supplementary concept word, rare disease supplementary concept word, unique identifier, synonyms] (78466)
16. (skinfold or skin fold thickness).mp. [mp=title, abstract, original title, name of substance word, subject heading word, floating sub-heading word, keyword heading word, organism supplementary concept word, protocol supplementary concept word, rare disease supplementary concept word, unique identifier, synonyms] (10959)
17. (body mass index or BMI).mp. [mp=title, abstract, original title, name of substance word, subject heading word, floating sub-heading word, keyword heading word, organism supplementary concept word, protocol supplementary concept word, rare disease supplementary concept word, unique identifier, synonyms] (279689)
18. 6 or 7 or 8 or 9 or 10 or 11 or 12 or 13 or 14 or 15 or 16 or 17 (608903)
19. (infan* adj10 #13).mp. [mp=title, abstract, original title, name of substance word, subject heading word, floating sub-heading word, keyword heading word, organism supplementary concept word, protocol supplementary concept word, rare disease supplementary concept word, unique identifier, synonyms] (4182)
20. ((baby or babies) adj10 #13).mp. [mp=title, abstract, original title, name of substance word, subject heading word, floating sub-heading word, keyword heading word, organism supplementary concept word, protocol supplementary concept word, rare disease supplementary concept word, unique identifier, synonyms] (551)
21. ((child* preschool or preschool child) adj10 #13).mp. [mp=title, abstract, original title, name of substance word, subject heading word, floating sub-heading word, keyword heading word, organism supplementary concept word, protocol supplementary concept word, rare disease supplementary concept word, unique identifier, synonyms] (2)
22. (early childhood adj10 #13).mp. [mp=title, abstract, original title, name of substance word, subject heading word, floating sub-heading word, keyword heading word, organism supplementary concept word, protocol supplementary concept word, rare disease supplementary concept word, unique identifier, synonyms] (60)
23. (young children adj10 #13).mp. [mp=title, abstract, original title, name of substance word, subject heading word, floating sub-heading word, keyword heading word, organism supplementary concept word, protocol supplementary concept word, rare disease supplementary concept word, unique identifier, synonyms] (91)
24. (kindergarten adj10 #13).mp. [mp=title, abstract, original title, name of substance word, subject heading word, floating sub-heading word, keyword heading word, organism supplementary concept word, protocol supplementary concept word, rare disease supplementary concept word, unique identifier, synonyms] (50)
25. ((children under 5 or children under five or under 5's) adj10 #13).mp. [mp=title, abstract, original title, name of substance word, subject heading word, floating sub-heading word, keyword heading word, organism supplementary concept word, protocol supplementary concept word, rare disease supplementary concept word, unique identifier, synonyms] (66)
26. 19 or 20 or 21 or 22 or 23 or 24 or 25 (4931)
27. (diabetes type 2 or type 2 diabetes or diabetes mellitus or insulin resistance syndrome* or insulin resistan* or hyperglycaemia or hypertension or arteriosclerosis or cardiovascular disease* or cardio vascular disease* or blood pressure or coronary heart disease* or metabolic syndrome* or dysmetabolic syndrome* or cardio metabolic disorder* or cardiometabolic disorder* or lipid profile or lipid metabolism or lipid* profile or glucose metaboli* disorder* or glucose intoleran* or glucose toleran* or obes* or hypertension or artheroscleros*).mp. (1781365)
28. (diabetes type 2 or type 2 diabetes).mp. [mp=title, abstract, original title, name of substance word, subject heading word, floating sub-heading word, keyword heading word, organism supplementary concept word, protocol supplementary concept word, rare disease supplementary concept word, unique identifier, synonyms] (126496)
29. diabetes mellitus.mp. [mp=title, abstract, original title, name of substance word, subject heading word, floating sub-heading word, keyword heading word, organism supplementary concept word, protocol supplementary concept word, rare disease supplementary concept word, unique identifier, synonyms] (433938)
30. insulin resistance syndrome*.mp. [mp=title, abstract, original title, name of substance word, subject heading word, floating sub-heading word, keyword heading word, organism supplementary concept word, protocol supplementary concept word, rare disease supplementary concept word, unique identifier, synonyms] (1746)
31. insulin resistan*.mp. [mp=title, abstract, original title, name of substance word, subject heading word, floating sub-heading word, keyword heading word, organism supplementary concept word, protocol supplementary concept word, rare disease supplementary concept word, unique identifier, synonyms] (98055)
32. hyperglycaemia.mp. [mp=title, abstract, original title, name of substance word, subject heading word, floating sub-heading word, keyword heading word, organism supplementary concept word, protocol supplementary concept word, rare disease supplementary concept word, unique identifier, synonyms] (10223)
33. hypertension.mp. [mp=title, abstract, original title, name of substance word, subject heading word, floating sub-heading word, keyword heading word, organism supplementary concept word, protocol supplementary concept word, rare disease supplementary concept word, unique identifier, synonyms] (485390)
34. arteriosclerosis.mp. [mp=title, abstract, original title, name of substance word, subject heading word, floating sub-heading word, keyword heading word, organism supplementary concept word, protocol supplementary concept word, rare disease supplementary concept word, unique identifier, synonyms] (70980)
35. cardiovascular disease*.mp. [mp=title, abstract, original title, name of substance word, subject heading word, floating sub-heading word, keyword heading word, organism supplementary concept word, protocol supplementary concept word, rare disease supplementary concept word, unique identifier, synonyms] (260492)
36. cardio vascular disease*.mp. [mp=title, abstract, original title, name of substance word, subject heading word, floating sub-heading word, keyword heading word, organism supplementary concept word, protocol supplementary concept word, rare disease supplementary concept word, unique identifier, synonyms] (705)
37. blood pressure.mp. [mp=title, abstract, original title, name of substance word, subject heading word, floating sub-heading word, keyword heading word, organism supplementary concept word, protocol supplementary concept word, rare disease supplementary concept word, unique identifier, synonyms] (442915)
38. coronary heart disease*.mp. [mp=title, abstract, original title, name of substance word, subject heading word, floating sub-heading word, keyword heading word, organism supplementary concept word, protocol supplementary concept word, rare disease supplementary concept word, unique identifier, synonyms] (50273)
39. metabolic syndrome*.mp. [mp=title, abstract, original title, name of substance word, subject heading word, floating sub-heading word, keyword heading word, organism supplementary concept word, protocol supplementary concept word, rare disease supplementary concept word, unique identifier, synonyms] (57036)
40. dysmetabolic syndrome*.mp. [mp=title, abstract, original title, name of substance word, subject heading word, floating sub-heading word, keyword heading word, organism supplementary concept word, protocol supplementary concept word, rare disease supplementary concept word, unique identifier, synonyms] (111)
41. cardio metabolic disorder*.mp. [mp=title, abstract, original title, name of substance word, subject heading word, floating sub-heading word, keyword heading word, organism supplementary concept word, protocol supplementary concept word, rare disease supplementary concept word, unique identifier, synonyms] (87)
42. cardiometabolic disorder*.mp. [mp=title, abstract, original title, name of substance word, subject heading word, floating sub-heading word, keyword heading word, organism supplementary concept word, protocol supplementary concept word, rare disease supplementary concept word, unique identifier, synonyms] (562)
43. lipid* profile.mp. [mp=title, abstract, original title, name of substance word, subject heading word, floating sub-heading word, keyword heading word, organism supplementary concept word, protocol supplementary concept word, rare disease supplementary concept word, unique identifier, synonyms] (22957)
44. lipid metabolism.mp. [mp=title, abstract, original title, name of substance word, subject heading word, floating sub-heading word, keyword heading word, organism supplementary concept word, protocol supplementary concept word, rare disease supplementary concept word, unique identifier, synonyms] (99667)
45. glucose metaboli* disorder*.mp. [mp=title, abstract, original title, name of substance word, subject heading word, floating sub-heading word, keyword heading word, organism supplementary concept word, protocol supplementary concept word, rare disease supplementary concept word, unique identifier, synonyms] (1189)
46. glucose intoleran*.mp. [mp=title, abstract, original title, name of substance word, subject heading word, floating sub-heading word, keyword heading word, organism supplementary concept word, protocol supplementary concept word, rare disease supplementary concept word, unique identifier, synonyms] (16545)
47. glucose toleran*.mp. [mp=title, abstract, original title, name of substance word, subject heading word, floating sub-heading word, keyword heading word, organism supplementary concept word, protocol supplementary concept word, rare disease supplementary concept word, unique identifier, synonyms] (59483)
48. obes*.mp. [mp=title, abstract, original title, name of substance word, subject heading word, floating sub-heading word, keyword heading word, organism supplementary concept word, protocol supplementary concept word, rare disease supplementary concept word, unique identifier, synonyms] (352263)
49. artheroscleros*.mp. [mp=title, abstract, original title, name of substance word, subject heading word, floating sub-heading word, keyword heading word, organism supplementary concept word, protocol supplementary concept word, rare disease supplementary concept word, unique identifier, synonyms] (149)
50. 28 or 29 or 30 or 31 or 32 or 33 or 34 or 35 or 36 or 37 or 38 or 39 or 40 or 41 or 42 or 43 or 44 or 45 or 46 or 47 or 48 or 49 (1781365)
51. 26 and 50 (337)
52. remove duplicates from 51 (334)
53. (infan* adj10 (body composition or body fat or fat percentage or fat % or lean mass or fat mass of fat free mass or muscle mass or grip strength or hand strength or antropometr* or skinfold or skinfold thickness or body mass index or BMI)).mp. [mp=title, abstract, original title, name of substance word, subject heading word, floating sub-heading word, keyword heading word, organism supplementary concept word, protocol supplementary concept word, rare disease supplementary concept word, unique identifier, synonyms] (3773)
54. ((baby or babies) adj10 (body composition or body fat or fat percentage or fat % or lean mass or fat mass of fat free mass or muscle mass or grip strength or hand strength or antropometr* or skinfold or skinfold thickness or body mass index or BMI)).mp. [mp=title, abstract, original title, name of substance word, subject heading word, floating sub-heading word, keyword heading word, organism supplementary concept word, protocol supplementary concept word, rare disease supplementary concept word, unique identifier, synonyms] (312)
55. (baby adj10 (body composition or body fat or fat percentage or fat % or lean mass or fat mass of fat free mass or muscle mass or grip strength or hand strength or antropometr* or skinfold or skinfold thickness or body mass index or BMI)).mp. [mp=title, abstract, original title, name of substance word, subject heading word, floating sub-heading word, keyword heading word, organism supplementary concept word, protocol supplementary concept word, rare disease supplementary concept word, unique identifier, synonyms] (131)
56. (babies adj10 (body composition or body fat or fat percentage or fat % or lean mass or fat mass of fat free mass or muscle mass or grip strength or hand strength or antropometr* or skinfold or skinfold thickness or body mass index or BMI)).mp. [mp=title, abstract, original title, name of substance word, subject heading word, floating sub-heading word, keyword heading word, organism supplementary concept word, protocol supplementary concept word, rare disease supplementary concept word, unique identifier, synonyms] (190)
57. ((child* preschool or preschool child) adj10 (body composition or body fat or fat percentage or fat % or lean mass or fat mass of fat free mass or muscle mass or grip strength or hand strength or antropometr* or skinfold or skinfold thickness or body mass index or BMI)).mp. [mp=title, abstract, original title, name of substance word, subject heading word, floating sub-heading word, keyword heading word, organism supplementary concept word, protocol supplementary concept word, rare disease supplementary concept word, unique identifier, synonyms] (670)
58. (early childhood adj10 (body composition or body fat or fat percentage or fat % or lean mass or fat mass of fat free mass or muscle mass or grip strength or hand strength or antropometr* or skinfold or skinfold thickness or body mass index or BMI)).mp. [mp=title, abstract, original title, name of substance word, subject heading word, floating sub-heading word, keyword heading word, organism supplementary concept word, protocol supplementary concept word, rare disease supplementary concept word, unique identifier, synonyms] (357)
59. (young children adj10 (body composition or body fat or fat percentage or fat % or lean mass or fat mass of fat free mass or muscle mass or grip strength or hand strength or antropometr* or skinfold or skinfold thickness or body mass index or BMI)).mp. [mp=title, abstract, original title, name of substance word, subject heading word, floating sub-heading word, keyword heading word, organism supplementary concept word, protocol supplementary concept word, rare disease supplementary concept word, unique identifier, synonyms] (269)
60. (kindergarten* adj10 (body composition or body fat or fat percentage or fat % or lean mass or fat mass of fat free mass or muscle mass or grip strength or hand strength or antropometr* or skinfold or skinfold thickness or body mass index or BMI)).mp. [mp=title, abstract, original title, name of substance word, subject heading word, floating sub-heading word, keyword heading word, organism supplementary concept word, protocol supplementary concept word, rare disease supplementary concept word, unique identifier, synonyms] (74)
61. (children under 5 adj10 (body composition or body fat or fat percentage or fat % or lean mass or fat mass of fat free mass or muscle mass or grip strength or hand strength or antropometr* or skinfold or skinfold thickness or body mass index or BMI)).mp. [mp=title, abstract, original title, name of substance word, subject heading word, floating sub-heading word, keyword heading word, organism supplementary concept word, protocol supplementary concept word, rare disease supplementary concept word, unique identifier, synonyms] (3)
62. (children under five adj10 (body composition or body fat or fat percentage or fat % or lean mass or fat mass of fat free mass or muscle mass or grip strength or hand strength or antropometr* or skinfold or skinfold thickness or body mass index or BMI)).mp. [mp=title, abstract, original title, name of substance word, subject heading word, floating sub-heading word, keyword heading word, organism supplementary concept word, protocol supplementary concept word, rare disease supplementary concept word, unique identifier, synonyms] (2)
63. (under 5's adj10 (body composition or body fat or fat percentage or fat % or lean mass or fat mass of fat free mass or muscle mass or grip strength or hand strength or antropometr* or skinfold or skinfold thickness or body mass index or BMI)).mp. [mp=title, abstract, original title, name of substance word, subject heading word, floating sub-heading word, keyword heading word, organism supplementary concept word, protocol supplementary concept word, rare disease supplementary concept word, unique identifier, synonyms] (0)
64. 53 or 54 or 57 or 58 or 59 or 60 or 61 or 62 or 63 (5194)
65. 50 and 64 (2219)
66. (diabetes type 2 or type 2 diabetes or diabetes mellitus or insulin resistance syndrome* or insulin resistan* or hyperglycaemia or hypertension or arteriosclerosis or cardiovascular disease* or cardio vascular disease* or blood pressure or coronary heart disease* or metabolic syndrome* or dysmetabolic syndrome* or cardio metabolic disorder* or cardiometabolic disorder* or lipid profile or lipid metabolism or lipid* profile or glucose metaboli* disorder* or glucose intoleran* or glucose toleran* or obes* or artheroscleros*).mp. (1781365)
67. ((infan* or baby or babies or child* preschool or preschool child or early childhood or young children or kindergarten* or children under 5 or children under five or under 5's) adj10 (body composition or body fat or fat percentage or fat % or lean mass or fat mass of fat free mass or muscle mass or grip strength or hand strength or anthropometr* or skinfold or skinfold thickness or body mass index or BMI)).mp. (5194)
68. 66 and 67 (2219)
69. ((infant* or baby or babies or child* preschool or preschool child or early childhood or young children or kindergarten* or children under 5 or children under five or under 5's) adj10 (percent fat or fat percent or densitometry)).mp. [mp=title, abstract, original title, name of substance word, subject heading word, floating sub-heading word, keyword heading word, organism supplementary concept word, protocol supplementary concept word, rare disease supplementary concept word, unique identifier, synonyms] (71)
70. 67 or 69 (5242)
71. 66 and 70 (2221)
72. limit 71 to english language (2110)
73. limit 72 to humans (1911)
74. limit 73 to yr="1990 -Current” (1817)
75. remove duplicates from 74 (1817)

## Appendix B

Detailed summary of included studies

**Table B1:** Full summary of included studies

Lead author, year, study design	Setting, sample size in analysis (%female)	Type of exposure	Age exposure assessed	Age outcome assessed (mean age)	Outcome measured	Key findings
High-income countries						
**Angbratt 2011**^(31)^ **Retrospective cohort**	Sweden, *n* 3579 (48 %)	PI, BMI	PI: birth, 1·5 years BMI: 2·5, 5, 7, 10 and 15 years	∼15 years (14·5 years)	Obesity (BMI > 30)	Moderate/small correlations was found between PI at birth, 1·5 years and BMI at 2·5 years with BMI at 15 years (r < 0·5). Strong correlation between BMI at 5, 7 and 10 years with BMI at 15 years (r > 0·5). Females: 5 years (r = 0·63;0·601–0·658), 7 years (r = 0·70; 0·675–0·723), 10 years (r = 0·81;0·793–0·826) Males: 5 years (r = 0·57; 0·538–0·600), 7 years (r = 0·68;0·655–0·704), 10 years (r = 0·79;0·772–0·807)
**Barker 2005,**^(32)^ **Birth cohort 1934–44**	Finland, *N* 444 (19·6 %) Clinical subset: *n* 2003	PI, BMI	Birth 11 years	∼37–64 years	Coronary events clinical subsample: fasting plasma glucose, insulin, sBP, TC	For both sexes fasting plasma glucose, insulin and sBP fell both with increasing birth weight and with increasing BMI at 2 years. TC was not associated with birth weight or BMI at 2 years. Females: BMI at 2 and 11 years are not associated with later coronary events. BMI at 2 and 11 yearsare associated with later coronary events in a simultaneous regression (*P* = 0·001 and *P* = 0·04, respectively). The HR associated with an increase in BMI of 1 sd were 0·62 (CI 0·46–0·82) at 2 years of age and 1·35 (CI 1·02–1·78) at 11 years. Males: Low BMI and PI at birth, and BMI at 1 and 2 years predicted later coronary events (all *P* < 0·001). BMI at 11 years is not associated with coronary events on its own’ however, in a simultaneous regression, low BMI at 2 years and high BMI at 11 were associated with coronary events (*P* < 0·001 and *P* = 0·05, respectively). The HR associated with an increase in BMI of 1 sd were 0·76 (CI 0·66–0·87) at 2 years of age and 1·14 (CI 1·00–1·31) at 11 years.
**East 2020,**^(33)^ **Randomised prospective cohort (Santiago Longitudinal Study)**	Chile, *n* 1000 (n/r)	BMI, WC	Birth, 3 months, 6 months, 1 years, 23 years	∼23 years	CM risk: sBP, dBP, fasting serum, total glucose, TG, TC, HDL-C, high sensitivity CRP, abdominal obesity, MetS	All results are presented as data from regression models (Y axis: mean BMI, X axis: age in years) in ‘risk present *v*. risk absent’ clusters. Hyperglycaemia: *slope 6mo-5* years : 0·05 *v*. −0·12, *intercept 5* years : 18·05 *v*. 16·78*, slope 5–23* years : 3·17 *v*. 2·93 High TG; *slope 6mo-5* years : −0·003 *v*. −0·13, *intercept 5* years : 17·82 *v*. 16·69*, slope 5–23* years : 3·76 *v*. 3·07. Low LDL-cholesterol: *slope 6mo-5* years : 0·04 *v*. −0·13, *intercept 5* years : 17·02 *v*. 16·56, *slope 5–23* years : 3·29 *v*. 2·99 Hypertension: *slope 6mo-5* years : 0·04 *v*. −.13*, intercept 5* years : 18·54 *v*. 16·66*, slope 5–23* years : 4·00 *v*. 3·09 Abdominal obesity: *slope 6mo-5* years : 0·05 *v*. −0·17, *intercept 5y:* 18·12 *v*. 16·25, *slope 5–23y:* 4·20 *v*. 2·70 MetS: *slope 6mo-5y:* 0·07 *v*. −0·13, *intercept 5y:* 18·44 *v*. 16·59*, slope 5–23y:* 4·22 *v*. 3·03 All CM risks were associated with a higher BMI at 5y.
**Eriksson 2003,**^(34)^ **Birth cohort 1934–44**	Finland, *n* 4515 (52·7 %)	PI, BMI	Birth, 3, 5, 7, 9, 11 years	∼56–66 years	Obesity (BMI > 30)	Children who became obese in adult life had above-average BMI at all ages from birth to 12y (all *P* < 0·001) Females: A PI > 18·5 at birth had an OR > 3 of becoming obese as adults *v*. those with a PI < 18·5. Males: A PI > 18·5 at birth had an OR > 4 of becoming obese as adults *v*. those with a PI < 18·5.
**Eriksson 2015,**^(35)^ **Birth cohort 1934–44**	Finland, *n* 13 345 (n/r) clinical subsample: *n* 2003 (n/r)	BMI	Birth-11 years	40 + years Clinical sunsample mean age: 62 years	T2D Clinical subsample: body composition and glucose tolerance	Among children with a BMI above the median at 11 years (group 1), each unit increase in BMI Z-score from birth to 7 years reduced the risk of T2D. After 7 years a higher BMI z-score was associated with increased risk of T2D. Among children with a BMI below the median at 11 years (group 2), each unit increase in BMI Z-score reduced the risk of T2D from birth to 11 years. In group 1, low BMI at birth and at 2 years and high BMI at 11 years were associated with T2D (OR 0·87;0·80, 0·95, OR0·86;0·78–0·94, OR 1·61;1·40, 1·83, respective years). In group 2, low BMI at birth was associated with T2D (OR:0·80;0·72–0·86). Females: Increased risk of T2D in girls with BMI above the median at 11 years (OR1·35;1·10, 1·65; *P* = 0·004) *v*. those below the median value. Males: Increased risk of T2D in both years with BMI above the median at 11 years (OR1·23;1·05, 1·44; *P* = 0·01) *v*. those below the median value.
**Evensen 2019,**^(36)^ **Prospective cohort (The Tromsø Study: Fit Futures)**	Norway, *n* 907 (48 %)	BMI	Birth-2 years, 6 years	∼15–20	Adiposity (DXA scan), overweight/obesity (BMI > 25)	Birth weight associated with FFMI. Females: Overweight/obesity at 2·5 years is associated with increased odds of higher FMI SDS at 15–20 years (OR 2·00; 1·03–3·89; *P* < 0·05) *v*. normal weight. Overweight/obesity at 6 years is associated with increased odds of having a WC > 80 cm at 15–20 years (OR 4·79; 3·05, 7·48; *P* < 0·001) *v*. normal weight. Males: Overweight/obesity at 6 years was associated with increased odds of having a WC > 94 cm (OR 5·56;3·25, 9·54; *P* < 0·001) and higher FMI SDS (OR 4·14; 2·41, 7·09; *P* < 0·001) at age 15–20 *v*. normal weight.
**Geserick 2018,**^(37)^ **Retrospective cohort**	Germany, 34 196 (n/r)	BMI	Birth-18 years	∼18 years	Obesity	Accelerated annual change in BMI SDS (≥ 0·2 to < 2·0) in children 2–6 years associated with overweight/obesity at 18 years (RR1·43; 1·35, 1·49) *v*. those with a stable BMI.
**Giudici, 2017,**^(38)^ **Retrospective cohort**	France, *n* 1919 (56·6 %)	BMI, WC	Birth-10 years	∼20–60 years (30·7 years)	WC, MetS: sBP, dBP, total glucose, TG, HDL-C, LDL-C	Adults with MetS presented higher BMI from age 4–6 years (*P* = 0·01) and 7–10 years (*P* < 0·001) compared to adults without MetS. Adults with high WC (men; > 94 cm, women; > 80 cm) presented higher BMI at all ages (p-global < 0·001). Adults with high TG concentrations presented higher BMI from age 1·5 years (p-global = 0·001). No association between low _a_HDL-C and BMI in childhood (p-global = 0·62). No association between hyperglycaemia and BMI in childhood (p-global = 0·23). No association between _a_BP and BMI in childhood (p-global = 0·18).
**Golab 2019,**^(39)^ **Birth cohort (Generation R Study)**	Netherlands, *n* 593 (n/r)	Skinfold thicknesses: biceps, triceps, suprailiacal and subscapular	1·5 months , 6 months, 2 years	∼10 years	BMI, adiposity (DXA): FMI, subcutaneous FMI, visceral FMI, pericardial FMI, liver fat fraction	Higher central-to-total FM ratio at 1·5 months associated with higher BMI (*P* < 0·05), FMI (*P* < 0·01) and subcutaneous FMI at age 10 (*P* < 0·01). Higher total subcutaneous FM at 6 months and 2 years associated with higher BMI, FMI and subcutaneous FM at age 10. All *P* < 0·01, except for BMI at 6 months (*P* < 0·05). No association between exposure and visceral fat, pericardial fat and liver fat (all *P* > 0·05).
**Graversen 2014,**^(40)^ **Birth cohort 1996**	Finland, *n* 2120 (51·4 %)	Weight, BMI	Weight: 5mo, 1 years BMI: 2–5 years	∼31 years	BMI, WC, MetS: TG, HDL-C, sBP, dBP, fasting glucose	Children with a BMI in the ≥ 50–< 75 and ≥ 95 percentile at age 3 had increased risk of MetS (RR1·6; 1·2–2·1 and RR1·9; 1·2–3·0 respectively) *v*. those in the ≥ 5–< 50 percentile. BMI at ≥ 95 percentile at 4 years and 5 years had increased risk of MetS at age 31 years (RR 2·4;1·6–3·5 and RR 2·5;1·7–3·8 respectively) *v*. those in the ≥ 5–< 50 percentile. Linear relationship with BMI from birth to 5 years with _a_BMI and _a_WC. BMI from 3 to 5 years is inversely associated with _a_HDL-C.
**Holland 1993,**^(41)^ **Birth cohort (NHSD)**	Great Britain, *n* 2830 (49·8 %)	BMI	2, 4, 6–7, 11 and 14 years	∼36 years	BMI, BP	Females: sBP: One unit increase in BMI SDS between birth and 7 years is associated with 1·4 mmHg rise (CI:0·7, 2·1) One unit increase in BMI SDS between 7 years and adolescence is associated with 1·0 mmHg rise (CI:0–2·0) dBP: One unit increase in BMI SDS between birth and 7 years is associated with 0·5 mmHg rise (CI:0, 1·1) One unit increase in BMI SDS between 7 years and adolescence is associated with 0·3 mmHg rise (CI:-0·5, 1·1) Males: sBP: One unit increase in BMI SDS between birth and 7 years is associated with 0·7 mmHg rise (CI:0, 1·4) One unit increase in BMI SDS between 7 years and adolescence is associated with 1·4 mmHg rise (CI:0·5, 2·3) dBP: 1 unit increase in BMI SDS between birth and 7 years is associated with 1·0 mmHg rise (CI:0·4–1·6) 1 unit increase in BMI SDS between 7 years and adolescence is associated with 1·7 mmHg rise (CI:0·9, 2·4)
**Howe 2010,**^(42)^ **Birth cohort (Avon)**	UK, *n* 5113 (52·8 %)	PI, BMI	Birth-15 years	∼15 years (15·5 years)	CVD risk: BP, HDL-C, LDL-C, TG, CRP, glucose, insulin	Little evidence of association between PI changed from 0 to 2 years and CVD risk at 15 years (*P*-values n/r). Associations between 1SD increase in BMI at 5–5·5, 7–8·5 and 8·5–10 years and several risk factors at 15 years (stronger associations for HDL-C, log TG, sBP) (*P*-values n/r).
**Howe 2014,**^(43)^ **Birth cohort (Avon)**	England, *n* 3154 (n/r)	BMI	Birth-10 years	∼17 years (17·8 years)	Brachial sBP, central sBP, dBP	For both sexes, a 1SD z-score increase in BMI at 7 and 10 years was associated with all three outcomes, increase in BP being higher at age 10 (all *P* < 0·001). All associations were stronger in males. No associations between BMI < 7 years and later BP. Highest BP was seen in those with a low birth weight as well as overweight or obesity at age 2 and 17. Females: Low BMI at birth associated with elevated brachial sBP at 17 years (*P* = 0·004), and with central sBP as well as dBP (*P* = 0·001 and *P* = 0·04 respectively). Males: Low BMI at birth associated with elevated brachial sBP (*P* = 0·04).
**Huang 2012,**^(44)^ **Birth cohort (Raine)**	Australia, *n* 1053 (n/r)	BMI, skinfold thickness	1–3, 5, 8, 10 and 14 years	∼17 years	CVD risk: BP, insulin, glucose, TG, BMI, TC, HDL-C, LDL-C, high-sensitive CRP	Females: BMI from 1 to 5 years was higher at each subsequent timepoint (all *P* ≤ 0·001) in high risk cluster *v*. low risk cluster. High-risk group showed greater abdominal, subscapular and suprailiac skinfold thicknesses and chest circumference from 1 to 5 years of age (all *P* < 0·05). Males: BMI at 3 years was higher in high-risk *v*. low-risk group (*P* ≤ 0·001). High-risk group showed greater abdominal, subscapular and suprailiac skinfold thicknesses and chest circumference from 3 to 5 years (all *P* ≤ 0·001).
**Johnson 2014,**^(45)^ **Birth cohort (NSHD)**	Great Britain, *n* 1273 (52·6 %)	BMI	Birth, 2, 4, 6–7, 11, 25 and 20 years	∼60–64	cIMT	Females: no statistical association, all *P* < 0·05. Males: 1 unit increase in z-score BMI at 4 years and 20 years increased odds of high cIMT (OR 1·26;1·03, 1·54; *P* = 0·03, OR1·28;1·02–1·61; *P* = 0·03, respectively) in the upper *v*. the 3 lower quartiles.
**Johnson 2017,**^(46)^ **Birth cohort (Fels Longitudinal study)**	USA, *n* 350 (52·6 %)	BMI	9 months	∼20–60 years	BMI, FMI, FFMI	One unit increase in infant BMI Z-score associated with BMI at 20 years (β = 0·70;0·31–1·09; *P* < 0·001). one unit increase in infant BMI Z-score associated with FFMI at 20 years (β = 0·75; 0·37–1·12; *P* < 0·001). One unit increase in infant BMI Z-score associated with FFMI at 30 years (β = 0·34; 0·12–0·56). No associations between infant BMI and body composition after age 30.
**Kwon 2017,**^(47)^ **Birth cohort (Iowa Fluoride Study)**	USA, *n* 1093 (n/r) Adiposity (DXA) subset at 8 and 19 years: *n* 495, *n* 314	BMI	1·5, 3, 6, 9 months, 1 years, 16, 20 months, 2 years	∼8, 19 years	Adiposity (DXA scan)	Trajectories of BMI were analysed, trends were identified, SP was divided into “consistently low BMI in childhood” (group 1), ‘steep increase in BMI second year of life’ (group 2), ‘steep increase in BMI the first year of life’ (group 3), ‘consistently high BMI in childhood’ (group 4) Females: Group and group 4 had higher FMI at age 8 years and 19 years than group 1 and group 2. *P*-values < 0·01 and 0·04, respectively. Males: no statistical association.
**Lagström 2008,**^(48)^ **Prospective randomised trial (STRIP)**	Finland, *n* 541 (n/r)	BMI	Birth, 7, 13 months, 2–13 years	∼13 years	Obesity (Cole’s cut-off point)	Females: Girls who were overweight at 13 years exceeded cut-off point for overweight (22·58 kg/m^3^) from age 5 years onward. Males: Boys who were overweight at 13 years exceeded cut-off point for overweight (21·91 kg/m^3^) from age 8 years onward.
**Lammi 2009,**^(49)^ **Case-control study**	Finland, *n* 128 (n/r)	BMI	Birth-11 years	∼15–39 years (34·5 years)	T2D diagnose	Increased risk (OR 1·87; 1·04, 3·37, *P* = 0·04) of T2D with a gain of 1kg/m^2^ in the minimum BMI at between 3 and 11 years old *v*. those who gained less than 1 kg/m^2^.
**Salonen 2009,**^(29)^ **Birth cohort 1934–44**	Finland, *n* 588 (n/r) Clinical subset: 2003 (n/r) * Normal weight SP in adulthood	BMI	Birth-11 years	∼56–70 (61·5 years)	BMI Clinical subsample: MetS	Females: No association was seen in women. Males: Inverse association between MetS and 1 sd z-score increase in BMI between ages 0–2 years (OR 0·53;0·33, 0·87).
**Skidmore 2007,**^(50)^ **Birth cohort (NSHD)**	Great Britain, *n* 2311 (n/r)	BMI	2 years, 4 years,7 years, 11 years, 15 years, 36 years	∼53 years	Lipid levels	Negative association between TC and 1 sd increase in BMI at age 2 and 4 years (*P* = 0·007 and 0·003, respectively). Positive associations between BMI at 36–56 and TC and LDL-C (all *P* < 0·05). All results are reported after adjusting for adult BMI, except for age 36 and 53. Females: Negative association between BMI from 7 to 15 years and HDL-C (regression coefficient-0·098; −0·139, −0·057; *P* < 0·001) Males: Negative association between BMI from 7 to 15 years and HDL-C (regression coefficient-0·044; −0·079, −0·010; *P* = 0·01)
**Ziyab 2014,**^(51)^ **Birth cohort (The Isle of weight birth cohort)**	UK, *n* 1240 (n/r)	BMI	1–2, 4 and 10 years	∼18 years	BP	Early persistent obesity trajectory (IOTF cut-offs for obesity were exceeded at age 4, 10 and 18 years) experienced higher sBP (mean difference 11·3 mmHg) and dBP (mean difference 12 mmHg) than the normal trajectory. All *P* < 0·001. Delayed overweight (IOTF thresholds for overweight were crossed at age 10 and 18 years) experienced higher sBP (mean difference 6·1 mmHg) and dBP (mean difference 5·5 mmHg) than the normal trajectory.

↑ = increased.

a: adult; BP: blood pressure; CI: confidence intervals; CM: cardiometabolic; cIMT: carotid intima-media thickness; CRP: c-reactive protein; dBP: diastolic blood pressure; DM; diabetes mellitus; DXA: dual energy X-ray absorptiometry; FFM: fat free mass; FFMI: fat free mass index; FM: fat mass; FMI: fat mass index; HDL-C: HDL-cholesterol; HOMA-IR; Homeostatic Model Assessment for Insulin Resistance; HR: hazard ratio; LBW: low birth weight; LDL-C: LDL-cholesterol; IGT; impaired glucose tolerance; INCAP: Institute of Nutrition of Central America and Panama Oriente; IOTF: International Obesity Taskforce; MetS: metabolic syndrome; n; sample size included in analysis; NHSD: National Survey of Health and Development Cohort; n/r; not reported; PBF: percentage body fat; PI: Ponderal index; RR: risk ratio; sBP: systolic blood pressure; SF: skinfold; SP: study population; STRIP: Special Turku Coronary Risk Factor Intervention Project; TC: total cholesterol; TG: total glucose; T2D: type 2 diabetes; WC: waist circumference; WHR; waist hip ratio.

## References

[1] Mendis S , Armstrong T , Bettcher D et al. (2014) Global Status Report On Noncommunicable Diseases 2014. Geneva: WHO.

[2] Ajay VS , Watkins DA & Prabhakaran D (2017) Relationships among major risk factors and the burden of cardiovascular diseases, diabetes, and chronic lung disease. In Cardiovascular, Respiratory, and Related Disorders, 3rd ed. [ D Prabhakaran , S Anand , TA Gaziano et al., editors]. Washington, DC: The World Bank. 30212084

[3] Branca F , Lartey A , Oenema S et al. (2019) Transforming the food system to fight non-communicable diseases. BMJ 364, l296–l296. 30692128 10.1136/bmj.l296 PMC6349221

[4] Vijayaraghavan K (1987) Anthropometry for assessment of nutritional status. Indian J Pediatr 54, 511–520. 3653956 10.1007/BF02749045

[5] Kerac M , McGrath M , Connell N et al. (2020) ‘Severe malnutrition’: thinking deeply, communicating simply. BMJ Global Health 5, e003023. 10.1136/bmjgh-2020-003023 PMC7677332 33208313

[6] Wells JCK (2019) Body composition of children with moderate and severe undernutrition and after treatment: a narrative review. BMC Med 17, 215. 31767002 10.1186/s12916-019-1465-8 PMC6878632

[7] Owino VO , Murphy-Alford AJ , Kerac M et al. (2019) Measuring growth and medium- and longer-term outcomes in malnourished children. Matern Child Nutr 15, 1–10. 10.1111/mcn.12790 PMC7199054 30690903

[8] Kumaran K , Lubree H , Bhat DS et al. (2020) Birth weight, childhood and adolescent growth and diabetes risk factors in 21-year-old Asian Indians: the Pune Children’s Study. J Dev Orig Health Dis 12, 1–10. 10.1017/S2040174420000707 PMC7616908 32753090

[9] Wells JCK & Fewtrell MS (2006) Measuring body composition. Arch Dis Child 91, 612–617. 16790722 10.1136/adc.2005.085522 PMC2082845

[10] Pullar J , Wickramasinghe K , Demaio AR et al. (2019) The impact of maternal nutrition on offspring’s risk of non-communicable diseases in adulthood: a systematic review. J Global Health 9, 20405. 10.7189/jogh.09.020405 PMC6790233 31656604

[11] Barouki R , Gluckman PD , Grandjean P et al. (2012) Developmental origins of non-communicable disease: implications for research and public health. Environ Health 11, 42. 22715989 10.1186/1476-069X-11-42 PMC3384466

[12] Forsén T , Eriksson JG , Tuomilehto J et al. (1999) Growth in utero and during childhood among women who develop coronary heart disease: longitudinal study. BMJ 319, 1403–1407. 10574856 10.1136/bmj.319.7222.1403 PMC28284

[13] Pineda E , Sanchez-Romero LM , Brown M et al. (2018) Forecasting future trends in obesity across Europe: the value of improving surveillance. Obes Facts 11, 360–371. 30308509 10.1159/000492115 PMC6257099

[14] Bogers RP , Bemelmans WJE , Hoogenveen RT et al. (2007) Association of overweight with increased risk of coronary heart disease partly independent of blood pressure and cholesterol levels: a meta-analysis of 21 cohort studies including more than 300 000 persons. Arch Intern Med 167, 1720–1728. 17846390 10.1001/archinte.167.16.1720

[15] Wells JC , Sawaya AL , Wibaek R et al. (2020) The double burden of malnutrition: aetiological pathways and consequences for health. Lancet 395, 75–88. 31852605 10.1016/S0140-6736(19)32472-9 PMC7613491

[16] Grey K , Gonzales GB , Abera M et al. (2021) Severe malnutrition or famine exposure in childhood and cardiometabolic non-communicable disease later in life: a systematic review. BMJ Global Health 6, e003161. 10.1136/bmjgh-2020-003161 PMC7949429 33692144

[17] Fabiansen C , Cichon B , Yaméogo CW et al. (2020) Association between admission criteria and body composition among young children with moderate acute malnutrition, a cross-sectional study from Burkina Faso. Sci Rep 10, 13266. 32764545 10.1038/s41598-020-69987-9 PMC7413376

[18] Lelijveld N , Musyoki E , Adongo SW et al. (2021) Relapse and post-discharge body composition of children treated for acute malnutrition using a simplified, combined protocol: a nested cohort from the ComPAS RCT. PLoS One 16, e0245477–e0245477. 33534818 10.1371/journal.pone.0245477 PMC7857614

[19] Wells JCK (2018) The capacity–load model of non-communicable disease risk: understanding the effects of child malnutrition, ethnicity and the social determinants of health. Eur J Clin Nutr 72, 688–697. 29748656 10.1038/s41430-018-0142-x

[20] Ford ND , Martorell R , Mehta NK et al. (2016) Life-course body mass index trajectories are predicted by childhood socioeconomic status but not exposure to improved nutrition during the first 1000 days after conception in guatemalan adults. J Nutr 146, 2368–2374. 27655759 10.3945/jn.116.236075 PMC5086792

[21] Ylihärsilä H , Kajantie E , Osmond C et al. (2007) Birth size, adult body composition and muscle strength in later life. Int J Obes 31, 1392–1399. 10.1038/sj.ijo.0803612 17356523

[22] Simmonds M , Burch J , Llewellyn A et al. (2015) The Use of Measures of Obesity in Childhood for Predicting Obesity and the Development of Obesity-Related Diseases in Adulthood: A Systematic Review and Meta-Analysis. Health Technology Assessment, vol. 19. York: Centre for Reviews and Dissemination, University of York; NIHR Journals Library. 10.3310/hta19430 PMC4781104 26108433

[23] Brisbois TD , Farmer AP & McCargar LJ (2012) Early markers of adult obesity: a review. Obes Rev 13, 347–367. 22171945 10.1111/j.1467-789X.2011.00965.x PMC3531624

[24] Singh AS , Mulder C , Twisk JWR et al. (2008) Tracking of childhood overweight into adulthood: a systematic review of the literature. Obes Rev 9, 474–488. 18331423 10.1111/j.1467-789X.2008.00475.x

[25] Park MH , Falconer C , Viner RM et al. (2012) The impact of childhood obesity on morbidity and mortality in adulthood: a systematic review. Obes Rev 13, 985–1000. 22731928 10.1111/j.1467-789X.2012.01015.x

[26] Yajnik CS & Yudkin JS (2004) The Y-Y paradox. Lancet 363, 163. 14726172 10.1016/S0140-6736(03)15269-5

[27] Moher D , Liberati A , Tetzlaff J et al. (2009) Preferred Reporting Items for Systematic Reviews and Meta-Analyses: the PRISMA Statement. PLoS Med 6, e1000097. 19621072 10.1371/journal.pmed.1000097 PMC2707599

[28] National Institute for Health and Clinical (2012) Methods for the Development of NICE Public Health Guidance, 3rd ed. London: NIHC. 27905711

[29] Salonen MK , Kajantie E , Osmond C et al. (2009) Childhood growth and future risk of the metabolic syndrome in normal-weight men and women. Diabetes Metab 35, 143–150. 19246227 10.1016/j.diabet.2008.10.004

[30] Corvalán C , Gregory CO , Ramirez-Zea M et al. (2007) Size at birth, infant, early and later childhood growth and adult body composition: a prospective study in a stunted population. Int J Epidemiol 36, 550–557. 17376801 10.1093/ije/dym010

[31] Angbratt M , Ekberg J , Walter L et al. (2011) Prediction of obesity from infancy to adolescence. Acta Paediatr 100, 1249–1252. 21592225 10.1111/j.1651-2227.2011.02326.x

[32] Barker DJP , Osmond C , Forsén TJ et al. (2005) Trajectories of growth among children who have coronary events as adults. N Engl J Med 353, 1802–1809. 16251536 10.1056/NEJMoa044160

[33] East P , Delker E , Blanco E et al. (2020) BMI trajectories from birth to 23 years by cardiometabolic risks in young adulthood. Obesity 28, 813–821. 32108435 10.1002/oby.22754 PMC7093235

[34] Eriksson J , Forsén T , Osmond C et al. (2003) Obesity from cradle to grave. Int J Obes 27, 722–727. 10.1038/sj.ijo.0802278 12833117

[35] Eriksson JG , Kajantie E , Lampl M et al. (2015) Trajectories of body mass index amongst children who develop type 2 diabetes as adults. J Intern Med 278, 219–226. 25683182 10.1111/joim.12354

[36] Evensen E , Emaus N , Furberg AS et al. (2019) Adolescent body composition and associations with body size and growth from birth to late adolescence. The Tromsø study: fit futures—a Norwegian longitudinal cohort study. Pediatr Obes 14, 1–13. 10.1111/ijpo.12492 30590874

[37] Geserick M , Vogel M , Gausche R et al. (2018) Acceleration of BMI in early childhood and risk of sustained obesity. N Engl J Med 379, 1303–1312. 30281992 10.1056/NEJMoa1803527

[38] Giudici KV , Rolland-Cachera MF , Gusto G et al. (2017) Body mass index growth trajectories associated with the different parameters of the metabolic syndrome at adulthood. Int J Obes 41, 1518–1525. 10.1038/ijo.2017.119 28529329

[39] Golab BP , Voerman E , van der Lugt A et al. (2019) Subcutaneous fat mass in infancy and abdominal, pericardial and liver fat assessed by magnetic resonance imaging at the age of 10 years. Int J Obes 43, 392–401. 10.1038/s41366-018-0287-7 30568271

[40] Graversen L , Sørensen TIA , Petersen L et al. (2014) Preschool weight and body mass index in relation to central obesity and metabolic syndrome in adulthood. PLoS One 9, e89986. 24595022 10.1371/journal.pone.0089986 PMC3940896

[41] Holland FJ , Stark O , Ades AE et al. (1993) Birth weight and body mass index in childhood, adolescence, and adulthood as predictors of blood pressure at age 36. J Epidemiol Community Health 47, 432–435. 8120494 10.1136/jech.47.6.432 PMC1059853

[42] Howe LD , Tilling K , Benfield L et al. (2010) Changes in ponderal index and body mass index across childhood and their associations with fat mass and cardiovascular risk factors at age 15. PLoS One 5, e15186. 21170348 10.1371/journal.pone.0015186 PMC2999567

[43] Howe LD , Chaturvedi N , Lawlor DA et al. (2014) Rapid increases in infant adiposity and overweight/obesity in childhood are associated with higher central and brachial blood pressure in early adulthood. J Hypertens 32, 1789–1796. 25023150 10.1097/HJH.0000000000000269 PMC4162319

[44] Huang R-C , Mori TA , Burrows S et al. (2012) Sex dimorphism in the relation between early adiposity and cardiometabolic risk in adolescents. J Clin Endocrinol Metab 97, E1014–22. 22442267 10.1210/jc.2011-3007

[45] Johnson W , Kuh D , Tikhonoff V et al. (2014) Body mass index and height from infancy to adulthood and carotid intima-media thickness at 60 to 64 years in the 1946 British Birth Cohort study. Arterioscler Thromb Vasc Biol 34, 654–660. 24458709 10.1161/ATVBAHA.113.302572 PMC3977342

[46] Johnson W , Choh AC , Lee M et al. (2017) Is infant body mass index associated with adulthood body composition trajectories? An exploratory analysis. Pediatr Obes 12, 10–18. 26756208 10.1111/ijpo.12100

[47] Kwon S , Janz KF , Letuchy EM et al. (2017) Association between body mass index percentile trajectories in infancy and adiposity in childhood and early adulthood. Obesity 25, 166–171. 27804242 10.1002/oby.21673 PMC5182145

[48] Lagström H , Hakanen M , Niinikoski H et al. (2008) Growth patterns and obesity development in overweight or normal-weight 13-year-old adolescents: the STRIP study. Pediatric 122, 14–16. 10.1542/peds.2007-2354 18829786

[49] Lammi N , Moltchanova E , Blomstedt PA et al. (2009) Childhood BMI trajectories and the risk of developing young adult-onset diabetes. Diabetologia 52, 408–414. 19130040 10.1007/s00125-008-1244-0

[50] Skidmore PML , Hardy RJ , Kuh DJ et al. (2007) Life course body size and lipid levels at 53 years in a British birth cohort. J Epidemiol Community Health 61, 215–220. 17325398 10.1136/jech.2006.047571 PMC2652912

[51] Ziyab AH , Karmaus W , Kurukulaaratchy RJ et al. (2014) Developmental trajectories of Body Mass Index from infancy to 18 years of age: prenatal determinants and health consequences. J Epidemiol Community Health 68, 934–941. 24895184 10.1136/jech-2014-203808 PMC4174013

[52] Bhargava SK , Sachdev HS , Fall CHD et al. (2004) Relation of serial changes in childhood body-mass index to impaired glucose tolerance in young adulthood. N Engl J Med 350, 865–875. 14985484 10.1056/NEJMoa035698 PMC3408694

[53] Fall CHD , Sachdev HS , Osmond C et al. (2008) Adult metabolic syndrome and impaired glucose tolerance are associated with different patterns of BMI gain during infancy: data from the New Delhi birth cohort. Diabetes Care 31, 2349–2356. 18835958 10.2337/dc08-0911 PMC2584194

[54] Krishnaveni G V , Veena SR , Srinivasan K et al. (2015) Linear growth and fat and lean tissue gain during childhood: associations with cardiometabolic and cognitive outcomes in adolescent Indian children. PLoS One 10, e0143231. 26575994 10.1371/journal.pone.0143231 PMC4648488

[55] Raghupathy P , Antonisamy B , Geethanjali FS et al. (2010) Glucose tolerance, insulin resistance and insulin secretion in young south Indian adults: relationships to parental size, neonatal size and childhood body mass index. Diabetes Res Clin Pract 87, 283–292. 20115937 10.1016/j.diabres.2009.11.015 PMC3428893

[56] Sachdev HS , Fall CHD , Osmond C et al. (2005) Anthropometric indicators of body composition in young adults: relation to size at birth and serial measurements of body mass index in childhood in the New Delhi birth cohort. Am J Clin Nutr 82, 456–466. 16087993 10.1093/ajcn.82.2.456

[57] Weitz CA , Friedlaender FY & Friedlaender JS (2014) Adult lipids associated with early life growth in traditional melanesian societies undergoing rapid modernization: a longitudinal study of the mid-20th century. Am J Phys Anthropol 153, 551–558. 24382639 10.1002/ajpa.22453

[58] Wells JCK (2000) A Hattori chart analysis of body mass index in infants and children. Int J Obes 24, 325–329. 10.1038/sj.ijo.0801132 10757626

[59] Gallagher D , Visser M , Sepúlveda D et al. (1996) How useful is body mass index for comparison of body fatness across age, sex, and ethnic groups? Am J Epidemiol 143, 228–239. 8561156 10.1093/oxfordjournals.aje.a008733

[60] Department of Health and Human Services & Centers For Disease Control (2011) Body Mass Index: Considerations for Practitioners. CDC. https://stacks.cdc.gov/view/cdc/25368 (accessed August 2020).

[61] Wells JCK (2009) Historical cohort studies and the early origins of disease hypothesis: making sense of the evidence: workshop on ‘Nutritional models of the developmental origins of adult health and disease’. Proc Nutr Soc 68, 179–188. 19245738 10.1017/S0029665109001086

[62] Wells JCK & Shirley MK (2016) Body composition and the monitoring of non-communicable chronic disease risk. Global Health Epidemiol Genom 1, e18. 10.1017/gheg.2016.9 PMC5870426 29868210

[63] Aihie Sayer A , Syddall HE , Dennison EM et al. (2004) Birth weight, weight at 1 year of age, and body composition in older men: findings from the Hertfordshire Cohort Study. Am J Clin Nutr 80, 199–203. 15213049 10.1093/ajcn/80.1.199

[64] Li H , Stein AD , Barnhart HX et al. (2003) Associations between prenatal and postnatal growth and adult body size and composition. Am J Clin Nutr 77, 1498–1505. 12791630 10.1093/ajcn/77.6.1498

[65] Singhal A , Wells J , Cole TJ et al. (2003) Programming of lean body mass: a link between birth weight, obesity, and cardiovascular disease? Am J Clin Nutr 77, 726–730. 12600868 10.1093/ajcn/77.3.726

[66] Johnson W , Bann D & Hardy R (2018) Infant weight gain and adolescent body mass index: comparison across two British cohorts born in 1946 and 2001. Arch Dis Child 103, 974–980. 29674515 10.1136/archdischild-2017-314079

[67] Sachdev HS , Fall CHD , Osmond C et al. (2005) Anthropometric indicators of body composition in young adults: relation to size at birth and serial measurements of body mass index in childhood in the New Delhi birth cohort. Am J Clin Nutr 82, 456–466. 16087993 10.1093/ajcn.82.2.456

[68] Kraja AT , Borecki IB , North K et al. (2006) Longitudinal and age trends of metabolic syndrome and its risk factors: the Family Heart Study. Nutr Metab 3, 41. 10.1186/1743-7075-3-41 PMC1697811 17147796

[69] Head T , Daunert S & Goldschmidt-Clermont PJ (2017) The aging risk and atherosclerosis: a fresh look at arterial homeostasis. Front Genet 8, 216. 29312440 10.3389/fgene.2017.00216 PMC5735066

[70] Olokoba AB , Obateru OA & Olokoba LB (2012) Type 2 diabetes mellitus: a review of current trends. Oman Med J 27, 269–273. 23071876 10.5001/omj.2012.68 PMC3464757

[71] Lucas A , Fewtrell MS & Cole TJ (1999) Fetal origins of adult disease-the hypothesis revisited. BMJ 319, 245–249. 10417093 10.1136/bmj.319.7204.245 PMC1116334

[72] Ajala O , Mold F , Boughton C et al. (2017) Childhood predictors of cardiovascular disease in adulthood. A systematic review and meta-analysis. Obes Rev 18, 1061–1070. 28545166 10.1111/obr.12561

[73] Umer A , Kelley GA , Cottrell LE et al. (2017) Childhood obesity and adult cardiovascular disease risk factors: a systematic review with meta-analysis. BMC Public Health 17, 1–24. 28851330 10.1186/s12889-017-4691-z PMC5575877

[74] Lloyd LJ , Langley-Evans SC & McMullen S (2012) Childhood obesity and risk of the adult metabolic syndrome: a systematic review. Int J Obes 36, 1–11. 10.1038/ijo.2011.186 PMC3255098 22041985

[75] Johnson W , Kuh D , Tikhonoff V et al. (2014) Body mass index and height from infancy to adulthood and carotid intima-media thickness at 60 to 64 years in the 1946 British Birth Cohort Study. Arterioscler Thromb Vasc Biol 34, 654–660. 24458709 10.1161/ATVBAHA.113.302572 PMC3977342

[76] Owen CG , Whincup PH , Orfei L et al. (2009) Is body mass index before middle age related to coronary heart disease risk in later life? Evidence from observational studies. Int J Obes 33, 866–877. 10.1038/ijo.2009.102 PMC2726133 19506565

[77] Wright CM , Cole TJ , Fewtrell M et al. (2022) Body composition data show that high BMI centiles overdiagnose obesity in children aged under 6 years. Am J Clin Nutr 116, 122–131. 34967839 10.1093/ajcn/nqab421 PMC9257461

[78] Ahrens W (2004) Commentary: socioeconomic status: more than a confounder? Int J Epidemiol 33, 806–807. 15155692 10.1093/ije/dyh203

[79] Sharp MK , Hren D & Altman DG (2018) The STROBE extensions: considerations for Development. Epidemiology 29, e53. 30052544 10.1097/EDE.0000000000000899 PMC7659437

[80] Thurstans S , Opondo C , Seal A et al. (2022) Understanding sex differences in childhood undernutrition: a narrative review. Nutrients 14, 948. 35267923 10.3390/nu14050948 PMC8912557

[81] Thurstans S , Opondo C , Seal A et al. (2020) Boys are more likely to be undernourished than girls: a systematic review and meta-analysis of sex differences in undernutrition. BMJ Global Health 5, e004030. 10.1136/bmjgh-2020-004030 PMC7745319 33328202

[82] WHO (2022) No WHO European Regional Obesity Report 2022. Copenhagen: WHO Regional Office for Europe.

